# CCL4‐mediated targeting of spleen tyrosine kinase (Syk) inhibitor using nanoparticles alleviates inflammatory bowel disease

**DOI:** 10.1002/ctm2.339

**Published:** 2021-02-17

**Authors:** Wenbin Gong, Jiafei Yu, Tao Zheng, Peizhao Liu, Fan Zhao, Juanhan Liu, Zhiwu Hong, Huajian Ren, Guosheng Gu, Gefei Wang, Xiuwen Wu, Yun Zhao, Jianan Ren

**Affiliations:** ^1^ School of Medicine, Southeast University, Research Institute of General Surgery Jinling Hospital Nanjing China; ^2^ Research Institute of General Surgery Jinling Hospital Nanjing China; ^3^ Department of General Surgery, BenQ Medical Center The Affiliated BenQ Hospital of Nanjing Medical University Nanjing China

**Keywords:** CCL4, inflammatory bowel disease, piceatannol, Syk

## Abstract

Inflammatory bowel disease (IBD) has emerged a global disease and the ascending incidence and prevalence is accompanied by elevated morbidity, mortality, and substantial healthcare system costs. However, the current typical one‐size‐fits‐all therapeutic approach is suboptimal for a substantial proportion of patients due to the variability in the course of IBD and a considerable number of patients do not have positive response to the clinically approved drugs, so there is still a great, unmet demand for novel alternative therapeutic approaches. Spleen tyrosine kinase (Syk), a cytoplasmic nonreceptor protein tyrosine kinase, plays crucial roles in signal transduction and there are emerging data implicating that Syk participates in pathogenesis of several gut disorders, such as IBD. In this study, we observed the Syk expression in IBD patients and explored the effects of therapeutic Syk inhibition using small‐molecule Syk inhibitor piceatannol in bone marrow–derived macrophages (BMDMs). In addition, due to the poor bioavailability and pharmacokinetics of small‐molecule tyrosine kinase inhibitors and superiority of targeting nanoparticles‐based drug delivery system, we herein prepared piceatannol‐encapsulated poly(lactic‐co‐glycolic acid) nanoparticles that conjugated with chemokine C–C motif ligand 4 (P‐NPs‐C) and studied its therapeutic effects in vitro in BMDMs and in vivo in experimental colitis model. Our results indicated that in addition to alleviating colitis, oral administration of P‐NPs‐C promoted the restoration of intestinal barrier function and improved intestinal microflora dysbiosis, which represents a promising treatment for IBD.

## Graphical abstract

1. The expression level of Syk increased in inflamed colon mucosa tissues from IBD patients and was associated with disease activity.

2. Piceatannol‐encapsulated poly(lactic‐co‐glycolic acid) nanoparticles that conjugated with chemokine CCL4 (P‐NPs‐C) alleviates experimental colitis.

3. Oral administration of P‐NPs‐C promotes the restoration of intestinal barrier function and improves intestinal microflora dysbiosis.

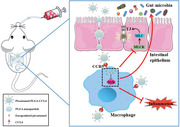

## 1 INTRODUCTION

Inflammatory bowel disease (IBD), typically including Crohn's disease (CD) and ulcerative colitis (UC), is a chronic and relapsing intestinal inflammatory disorder.^1^, ^2^ IBD has become a global disease with accelerating incidence in newly industrialized countries where societies have become more Westernized at the turn of the 21st century.^3^ The rising incidence of IBD is accompanied by higher morbidity, mortality, and substantial healthcare system costs.^4^ Although with unclear etiology, therapeutic strategies for patients with IBD at present mainly target mediators of inflammation to limit or suppress proinflammatory processes.^5^, ^6^ Typical examples are steroids, suppression of lymphocytes by thiopurines, or antibodies against cytokines such as tumor necrosis factor, integrin, and interleukin 12/23.^7^ Use of these agents is associated with restoration of mucosal functions, such as immune homeostasis and barrier function, and maintenance of mucosal healing, which emerges as the major long‐term therapeutic goal of IBD.^8^, ^9^ However, there are often patients who do not respond to these medications and the effects on maintaining remission of these medications are only temporary.

Our group and others have demonstrated that spleen tyrosine kinase (Syk) plays an essential role in the pathogenesis of IBD in recent years.^10^, ^11^, ^12^ Syk is a cytoplasmic nonreceptor protein tyrosine kinase that exists in both hematopoietic cells and nonhematopoietic cells.^13^ Activation of Syk in these cells can modulate intestinal mucosal immune response by driving inflammatory signaling pathways, thus regulating gut homeostasis.^14^ There are emerging data from both experimental and clinical studies demonstrating that therapeutic inhibition of Syk mitigates intestinal inflammation by reducing production of cytokines and chemokines in IBD.^15^, ^16^, ^17^ However, whether Syk has other pathogenic mechanisms in IBD (e.g., affecting intestinal barrier function or intestinal microflora) and whether inhibition of Syk relieves colitis by improving such effects is not entirely clear.

In addition, due to the poor bioavailability and pharmacokinetics of small‐molecule tyrosine kinase inhibitors,^18^ it is necessary to exploit novel drug delivery systems that are able to deliver drugs exclusively to the inflamed regions of intestine. One of the most promising carriers is poly(lactic‐co‐glycolic acid) (PLGA)‐derived nanoparticles that are commonly used as drug carriers in multiple biomedical applications.^19^, ^20^ Such nanoparticles‐based carrier system can assist in the loaded drug accumulation particularly at inflamed areas because of the growing permeability of lesions. Due to the superior ability to execute precise control over small quantities of fluids within integrated flow channels,^21^ a microfluidic approach was used in this paper for generating the Syk inhibitor piceatannol‐encapsulated PLGA nanoparticles. In addition, in order to improve the active targeting of nanoparticles, the chemokine C–C motif ligand 4 (CCL4) was conjugated with the piceatannol‐encapsulated PLGA nanoparticles to form piceatannol–PLGA–CCL4 nanoparticles (P‐NPs‐C). CCL4 can bind to its receptor CCR5 (C–C motif chemokine receptor 5), which is expressed on surface of most immune cells, especially on macrophages.

In this study, we observed the Syk expression levels in IBD patients and investigated the effects of therapeutic Syk inhibition on inflammatory macrophages by using small‐molecule Syk inhibitor piceatannol. We thereafter prepared P‐NPs‐C and investigated therapeutic efficacy in bone marrow–derived macrophages (BMDMs) and experimental colitis model. Our results indicate that in addition to alleviating colitis, P‐NPs‐C can promote the restoration of intestinal barrier function and improve intestinal microflora dysbiosis, which represents a promising treatment for IBD.

## 2 MATERIALS AND METHODS

### 2.1 Patients

The inflamed colon mucosa and uninflamed controls were taken during routine ileocolonoscopy from active IBD patients or healthy controls in Jinling Hospital. IBD patients were diagnosed based on the standard clinical, endoscopic, histologic, and radiologic criteria.^2^ Medical records were carefully scanned to classify the characteristics of the disease and the location of lesions was recorded on the basis of Montreal classification. Modified Mayo score, which includes stool frequency, rectal bleeding, endoscopic findings, and global assessment of a physician, was employed to assess the clinical activity of patients with UC. In particular, the Modified Mayo score ≤2 was defined as clinical remission, 3–5 mild activity, 6–10 moderate activity, and 11–12 severe active disease. In total, 13 patients with active UC were enrolled in our study. Correspondingly, the simple Crohn's disease activity index (CDAI) score, which accounts for general condition, presence of abdominal pain, abdominal mass, stool frequency, and disease complications, was used to assess the degree of clinical activity of patients with CD. A simple CDAI score ≤4 was considered as clinical remission, 5–7 mild activity, 8–16 moderate activity, and >16 severe active disease. Altogether, 17 patients with active CD were included. Finally, 10 age‐ and sex‐matched healthy controls who did not suffer from any gastrointestinal disorders or autoimmune diseases were recruited from the physical examination center in Jinling Hospital. More details of patients are shown in Table S1.

The collection of human specimens was approved by the institutional review board at Jinling Hospital (2018GKJDWLS‐03‐032). Written informed consent from each participant was obtained.

### 2.2 Cell line culture

The human monocyte THP‐1 cell line and human colorectal cancer cell line Caco‐2 were purchased from American Type Culture Collection (ATCC, Manassas, USA). High‐density Caco‐2 cells were grown on transwell filters (Corning Inc., Tewksbury, USA) and used for transepithelial electrical resistance (TEER) measurements and biochemical studies.

### 2.3 Piceatannol–PLGA–CCL4 nanoparticles preparation

The double‐emulsion microfluidic system was employed to fabricate the piceatannol‐encapsulated PLGA nanoparticles. Two coaxially assembled capillaries were placed on a glass slide and the connection spot was fixed by epoxy resin. The PLGA solution dissolved in dichloromethane (40 mg/ml) was employed as the outer phase and piceatannol (Selleck chemicals, USA) was dissolved in Ethanol (180 mM) for the inner phase. The round capillaries (World Precision Instrument, Inc., Shanghai, China) were tapered to desired diameters with a micropipette puller (Sutter Instrument, Inc., USA). The tapered round capillaries were installed into square capillaries with an inner dimension of 1.0 mm. The inner phase solution could be wrapped by outer phase in the microfluidic channel and monodispersed droplets of different sizes can be formed that were controlled by different flow rates of phases. The nanosized droplets could be obtained directly through the extrusion of emulsion droplets formed on microfluidics chip via the filter (0.2 μm).

CCL4 ligands (Sigma–Aldrich) were conjugated on piceatannol‐encapsulated PLGA nanoparticles through copper‐free click chemistry. Dibenzocyclooctyne‐S‐S‐*N*‐hydroxysuccinimidyl ester in DMSO (10 mg/ml) was added into nanoparticle emulsion droplets at a 10:1 molar ratio. NHS‐PEG4‐Azide in DMSO (40 mg/ml) was added into CCL4 solution at a 10:1 molar ratio. Both reactions took place in an ice bath for 1 h. The conjugation between dibenzocyclooctyne‐modified piceatannol‐encapsulated PLGA nanoparticles and azide‐modified CCL4 was reacted at 4°C overnight at a 1:1 molar ratio to form P‐NPs‐C. Followed by drying under vacuum and washing three times, the P‐NPs‐C were lyophilized to the dry powder.

### 2.4 Physicochemical characterization determination

The nanoparticles’ average size and size distribution of P‐NPs‐C were determined by dynamic light scattering, and zeta potential was measured with a Nano ZS Zetasizer (Malvern Instruments Ltd., Malvern, UK). The surface and internal morphology of P‐NPs‐C were observed by scanning electron microscope (SEM).

To conduct the drug release and uptake studies of P‐NPs‐C, the BMDMs were prepared in 12‐well plates and P‐NPs‐C were added in the cells. At time intervals of 0, 8, 16, 24, 32, 40, and 48 h, 100 μl of samples were collected and replaced by an equal volume of the medium. The amount of drug release was determined based on the standard curve that was measured with high‐performance liquid chromatography. The drug uptake was measured as the difference between the total drug and drug in the medium at multiple time points. The curves were plotted in terms of percentages of cumulative drug release and total drug uptake.

### 2.5 In vitro efficacy studies in BMDMs, THP‐1 cells, and Caco‐2 cells

For in vitro efficacy studies, BMDMs or THP‐1 cells were prepared in 12‐well plates and stimulated by IFN‐γ (20 ng/ml) and different concentrations of free drug piceatannol (25, 50, and 75 μM) or P‐NPs‐C for 48 h. Afterward, BMDMs were collected for immunofluorescence staining and lysed for western blot or real‐time PCR analysis. For assessing the efficacy of Syk inhibitor on intestinal epithelial barrier, a 12‐well transwell system was used. Caco‐2 cells were plated on the upper chamber and the conditioned media from BMDMs after aforementioned stimulation were filled in the bottom chamber. After 48 h of stimulation, tight junction proteins of Caco‐2 monolayers in the upper chamber were evaluated by immunofluorescence staining and western blot. In selected experiment, Caco‐2 cells were treated with a myosin light chain kinase (MLCK) inhibitor (ML‐7, 40 μM, Selleck Chemicals, USA).

### 2.6 Nitric oxide release bioassay

BMDMs were prepared in 12‐well plates and 20 ng/ml IFN‐γ was added in each well with multiple concentrations of Syk inhibitor piceatannol (0, 25, 50, and 75 μM). After 48 h of incubation, the absorbance at 540 nm was measured by a microplate reader.

### 2.7 Experimental models of colitis

The C57BL/6 male mice were obtained from Model Animal Research Center of Nanjing University. Animal care and use were approved by Jinling Hospital Animal Care Committee. Experimental model of acute colitis was induced as described before.^12^ In selected experiments, nanoparticles were orally administered in the hydrogel system comprising alginate/chitosan each day until day 8. Hydrogel (alginate/chitosan) could assist P‐NPs‐C in passing through stomach and small intestine smoothly and releasing the nanoparticles at colon after its degradation.

### 2.8 In vitro cellular uptake of P‐NPs‐C

The analysis of cellular uptake of nanoparticles was carried out using DiL‐loaded P‐NPs‐C by fluorescence microscopy imaging. BMDMs were prepared in six‐well plates and P‐NPs‐C/DiL were added in the cells and incubated for time intervals of 1, 2, 3, 4, and 8 h. BMDMs in the absence of P‐NPs‐C/DiL were used as control. Images were observed and analyzed by a fluorescence microscopy (NIKON Eclipse Ts2‐FI, Japan).

### 2.9 In vivo cellular internalization of P‐NPs‐C

Mice with 2.5% DSS‐induced experimental colitis were orally administered with P‐NPs‐C‐embedded hydrogel at day 8. After 2, 8, and 14 h of administration, mice were euthanized and the alimentary tract was collected. The fluorescence intensities of images were captured with an Odyssey CLX Infrared Imaging System (Gene Company Limited, China). In selected experiments, fluorescent lipophilic dye, 1,1′‐dioctadecyl‐3,3′,3′‐tetramethylindotricarbocyanine iodide (DiR), or 3,3′‐dioctadecyloxacarbocyanine perchlorate (DiO) loaded P‐NPs‐C were constructed by the same aforesaid microfluidic procedures and colon tissues of mice were collected for fluorescence staining.

### 2.10 Evaluation of inflammation in vivo

The intestinal inflammation in vivo was evaluated by IVIS imaging systems using XenoLight RediJect Inflammation Probe that was conveniently used to investigate activity of myeloperoxidase. The XenoLight RediJect probe (150 μl/mouse, 40 mg/ml) was injected intraperitoneally 5 min before imaging and bioluminescence signals in regions of interest were used for statistical analysis.

### 2.11 Quantification of lipocalin‐2

Lipocalin‐2 (Lcn‐2) levels from fresh feces of mice were evaluated via the Lipocalin‐2 ELISA kit (ab119601, Abcam).

### 2.12 In vivo intestinal permeability assay

Mice were fasted for 4 h but allowed free access to water and then given an oral gavage with 40 mg/100 g body weight of FITC‐4 kDa Dextran (Sigma‐Aldrich). Five hours postgavage, serum was collected from the mice by intracardiac puncture. Fluorescence intensity was detected at excitation wavelengths of 485 nm and emission wavelengths of 528 nm. FITC–Dextran concentrations were obtained by conversion from standard curves.

### 2.13 Histology, immunohistochemistry, immunofluorescence, and Alcian Blue staining

Histology, immunohistochemistry, and immunofluorescence were carried out as we described before.^12^ Primary antibodies CD45 monoclonal antibody (ab208022, Abcam), F4/80 polyclonal antibody (ab100790, Abcam), CD3 monoclonal antibody (ab135372, Abcam), and MPO monoclonal antibody (ab208670, Abcam) were used for immunohistochemistry. Antibodies p‐Syk (ab62338, Abcam), p‐Syk (ab63515, Abcam), F4/80 polyclonal antibody (ab100790, Abcam), ZO‐1 (ab221547, Abcam), Occludin (ab216327, Abcam), and EpCAM (ab20160, Abcam) were employed for immunofluorescence. For mucus evaluation, slides were stained with Alcian Blue (AB; Sigma Aldrich) solution, pH 2.5, for 5 min and washed under running tap water.

### 2.14 Western blot

The tissue and cell samples were lysed in RIPA buffer supplemented with protease inhibitor cocktail and phosphatase inhibitor cocktail (Jiangsu KeyGEN BioTECH, Nanjing, China) and the final concentration of lysates was adjusted to 1.5 μg/μl. When running western blots, the sample volume of each well was 10 μl, that is, 15 μg of total protein (15‐well electrophoresis comb, 1.5 mm). Syk (ab40781, Abcam) and p‐Syk (ab58575, Abcam) antibodies for human tissues and Syk (ab40781, Abcam), p‐Syk (ab58575, Abcam), MLCK (ab76092, Abcam), MLC (ab92721, Abcam), p‐MLC (ab2480, Abcam), ZO‐1 (ab96587, Abcam), and Occludin (ab216327, Abcam) antibodies for mouse tissues were used. The protein bands were visualized by a ChemiDoc Touch Imaging System (Bio‐Rad, USA) and quantified by the Image Lab software. Results were normalized to the GAPDH internal control.

### 2.15 Statistical analysis

The graphs and statistical analysis were carried out using GraphPad Prism software, version 7. The significance between groups was assessed by unpaired Student's *t*‐test or one‐way analysis of variance. Nonparametric Wilcoxon test was used for pairwise comparisons between UC and CD patients before and after medication. Quantitative data were shown as mean ± standard deviation (SD). The differences were considered significant at *P* < 0.05 (*< 0.05; **< 0.01; ***< 0.001).

Methods for BMDM preparation, biocompatibility assays, TEER and paracellular FD‐4 flux measurements, quantitative real‐time PCR, flow cytometry, and effects of P‐NPs‐C on intestinal microbiota are provided in the Supporting Information.

## 3 RESULTS

### 3.1 Increased Syk expression in inflamed colon mucosa from IBD patients

**FIGURE 1:**
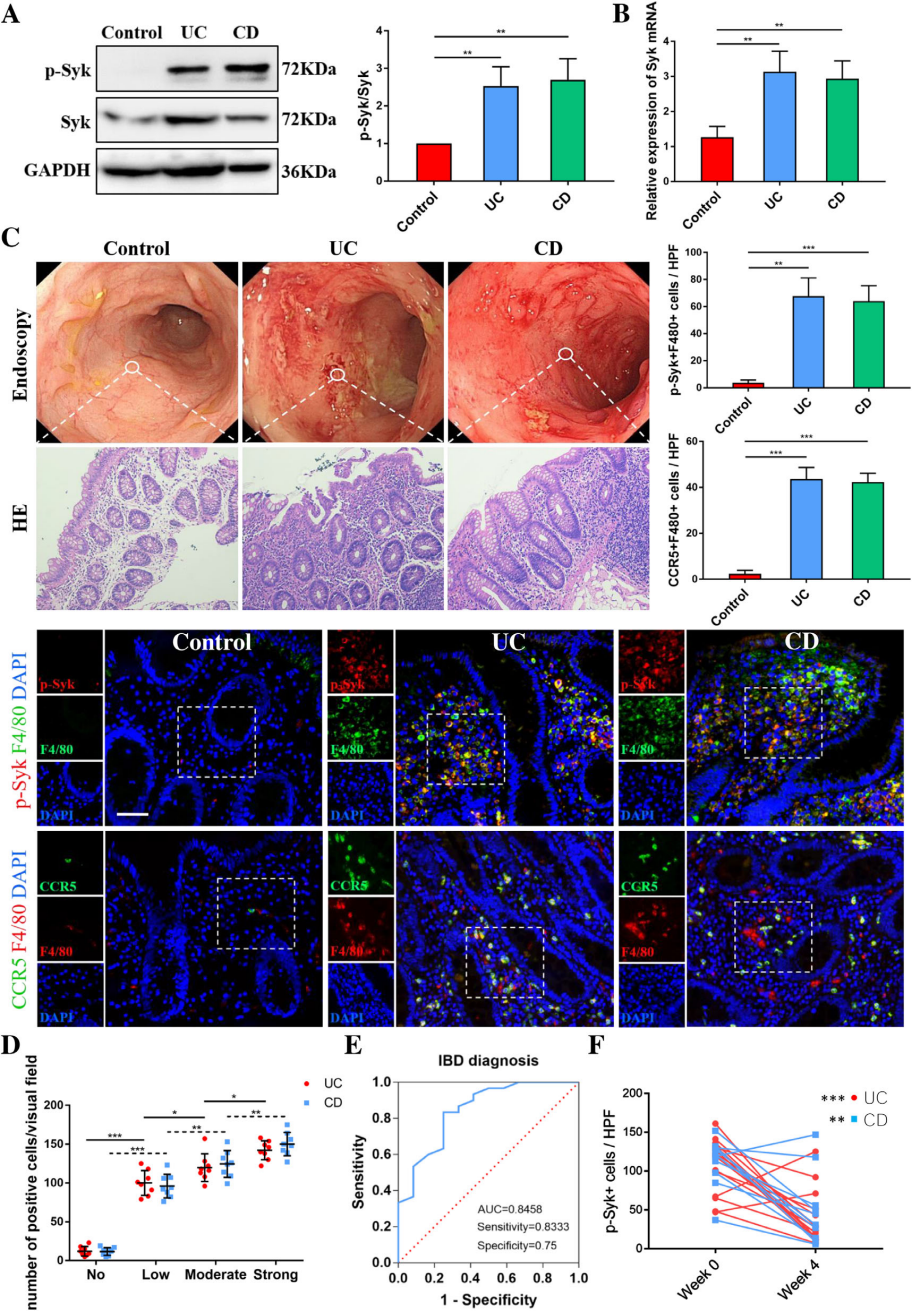
Elevated expression of Syk in the colon mucosal biopsies of active IBD patients. Syk expression of colonic biopsies from controls and active IBD patients were determined by western blot (A) and PCR (B) (*n* = 6/group). (C) Sections of colonic biopsies from endoscopy of controls or individuals with active IBD were analyzed for phosphorylated Syk (p‐Syk) and CCR5 by immunofluorescence. Nuclei were counterstained with DAPI (*n* = 7/group). (D) Phosphorylated Syk expression levels in colonic mucosa of IBD patients significantly correlated with disease activity. (E) Receiver operating characteristic curve for individual p‐Syk to separate IBD patients from healthy controls. (F) Phosphorylated Syk expression levels of IBD patients at week 0 versus the week 4 after treatment. Scale bars = 50 μm. Data were shown as mean values ± SD. *P < 0.05, **P < 0.01, and ***P < 0.001

To explore the role of Syk in the pathogenesis of IBD, we examined Syk expression in colon biopsies of IBD patients taken from ileocolonoscopy. Strikingly, an increased Syk phosphorylation (p‐Syk) in colonic mucosa of IBD was observed by western blot (Figure 1A). We also found elevated mRNA levels of Syk in IBD compared with healthy controls (Figure 1B). Fluorescence co‐staining of p‐Syk and F4/80 demonstrated an obvious Syk phosphorylation in the cytoplasm of F4/80+ macrophages within the mucosa lamina propria of IBD patients (Figure 1C). In addition, we found that the expression levels of p‐Syk were associated with the activity of disease and significantly higher expression of p‐Syk was found in samples from IBD patients with strong inflammation (Figure 1D). Interestingly, the analysis of receiver operating characteristic curve revealed that p‐Syk levels in colonic mucosa had a significant area under the curve of 0.8458 with 83.33% sensitivity and 75.00% specificity in diagnosing IBD (Figure 1E). To further explore the changing level of p‐Syk, colonic mucosa samples of 10 UC and 14 CD patients were collected after 4 weeks of medication, and the average p‐Syk levels in colonic mucosa decreased significantly after drug therapy (Figure 1F). Taken together, these results suggested that Syk played an indispensable role in the pathogenesis of IBD and it might emerge as a potential therapeutic target.

### 3.2 Piceatannol inhibited Syk activation‐induced inflammatory response and epithelial barrier dysfunction

**FIGURE 2:**
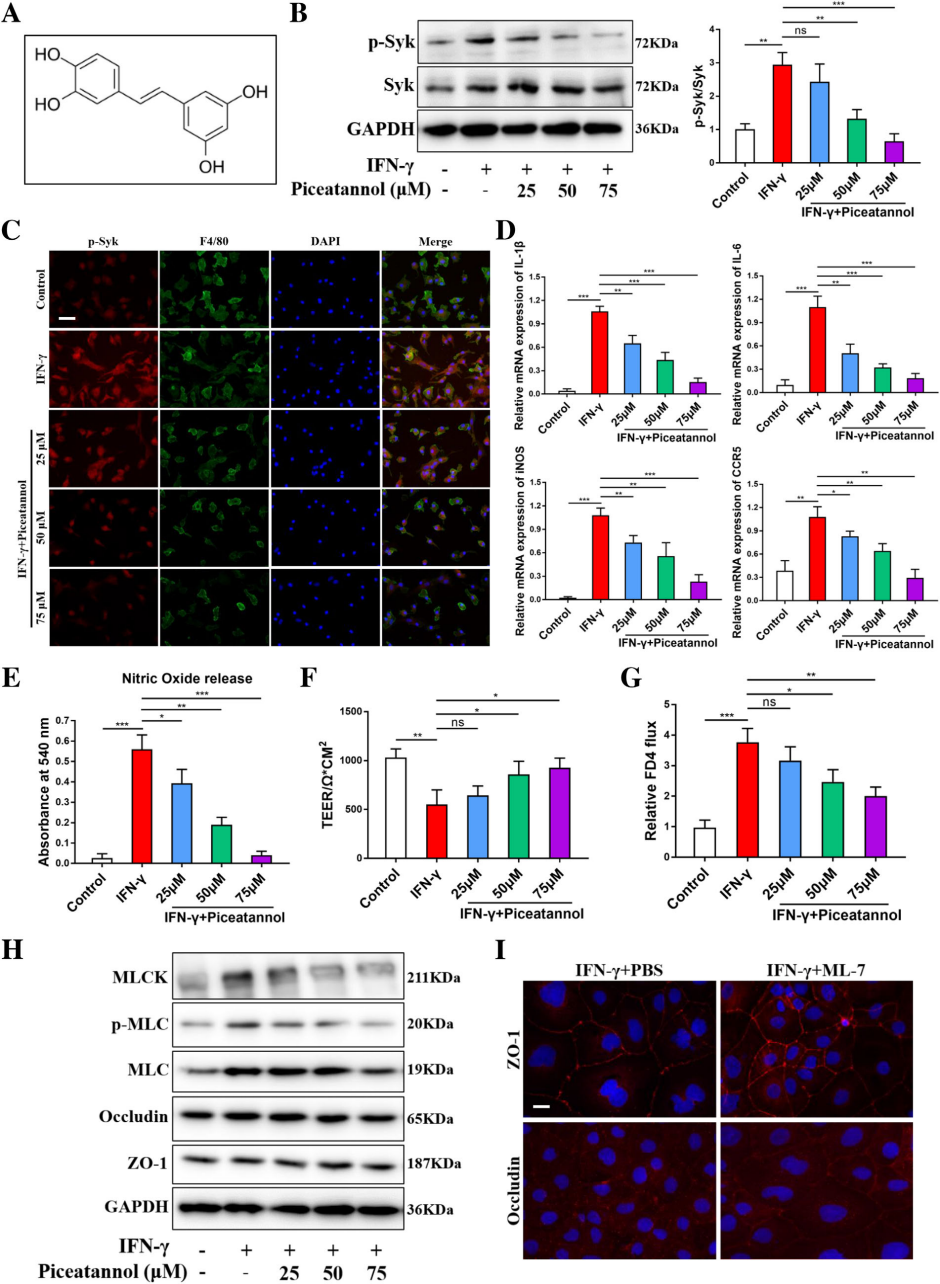
Piceatannol inhibited Syk activation‐induced inflammatory response and epithelial barrier dysfunction in vitro. (A) Structure of piceatannol, a Syk inhibitor. Protein expression of p‐Syk in bone marrow‐derived macrophages (BMDMs) after incubation with medium alone and 20 ng/ml IFN‐γ without and with piceatannol (25, 50, and 75 μM) was determined by western blot (B) and immunofluorescence staining (C). Scale bars = 100 μm. (D) Gene expression of inflammatory markers IL‐1β, iNOS, IL‐6, and CCR5 in BMDMs after incubation with medium alone or IFN‐γ stimulus with piceatannol (0, 25, 50, and 75 μM). (E) Nitric oxide (NO) release as measured in the culture supernatant of BMDMs. Syk activation resulted in a remarkable injury to monolayer barrier and piceatannol induced a concentration‐dependent recovery in monolayer barrier function by increasing TEER (F) and decreasing FD‐4 flux (G). (H) Piceatannol concentration dependently downregulated the protein levels of MLCK and the phosphorylation of MLC. (I) MLCK inhibitor ML‐7 ameliorated IFN‐γ‐induced irregular undulations of ZO1 and Occludin in Caco‐2 cells. Scale bars = 10 μm. Data were shown as mean values ± SD. *P < 0.05, **P < 0.01, ***P < 0.001, and ns, no significance

Owing to the upregulated expression of Syk and main location in macrophages of colonic lamina propria of IBD patients, we investigated the implication of this tyrosine kinase in BMDMs. Piceatannol (Figure 2A), a selective Syk inhibitor, was used to inhibit Syk signaling pathway. We examined the phosphorylation of Syk in BMDMs with stimulation of INF‐γ and observed that increased concentration of piceatannol was accompanied with decreased Syk phosphorylation (Figure 2B). Consistently, fluorescence staining of p‐Syk in BMDMs presented a similar result (Figure 2C). Afterward, the effects of piceatannol on Syk phosphorylation‐mediated functional activation were explored. We observed that piceatannol concentration dependently reduced IFN‐γ‐induced inflammatory gene expression, that is, IL‐1β, iNOS, IL‐6, CCR5, and nitric oxide release in BMDMs (Figures 2D and 2E).

To explore whether the phosphorylation of Syk affected intestinal barrier function, a Caco‐2 monolayer barrier function assay was carried out. Syk activation resulted in a remarkable injury to monolayer barrier, whereas piceatannol could induce a concentration‐dependent recovery in monolayer barrier function, which was reflected by increasing TEER and decreasing FD‐4 flux (Figure 2F,G). As MLCK‐mediated MLC phosphorylation has been shown to contribute to barrier dysregulation,^22^ we next detected whether Syk could regulate MLCK and p‐MLC. Piceatannol concentration dependently downregulated the protein levels of MLCK and the phosphorylation of MLC, but had no influence on tight junction proteins Occludin or ZO‐1 (Figure 2H). To confirm the effect of MLCK/p‐MLC on barrier function, Caco‐2 cells were stimulated with IFN‐γ with or without the MLCK inhibitor ML‐7. ML‐7 ameliorated IFN‐γ‐induced irregular undulations of ZO1 and Occludin (Figure 2I). Collectively, these data indicated that inhibition of Syk reduced inflammation and promoted barrier function recovery in vitro; hence, Syk inhibitor could be a promising treatment for IBD.

### 3.3 Characterization of P‐NPs‐C

**FIGURE 3:**
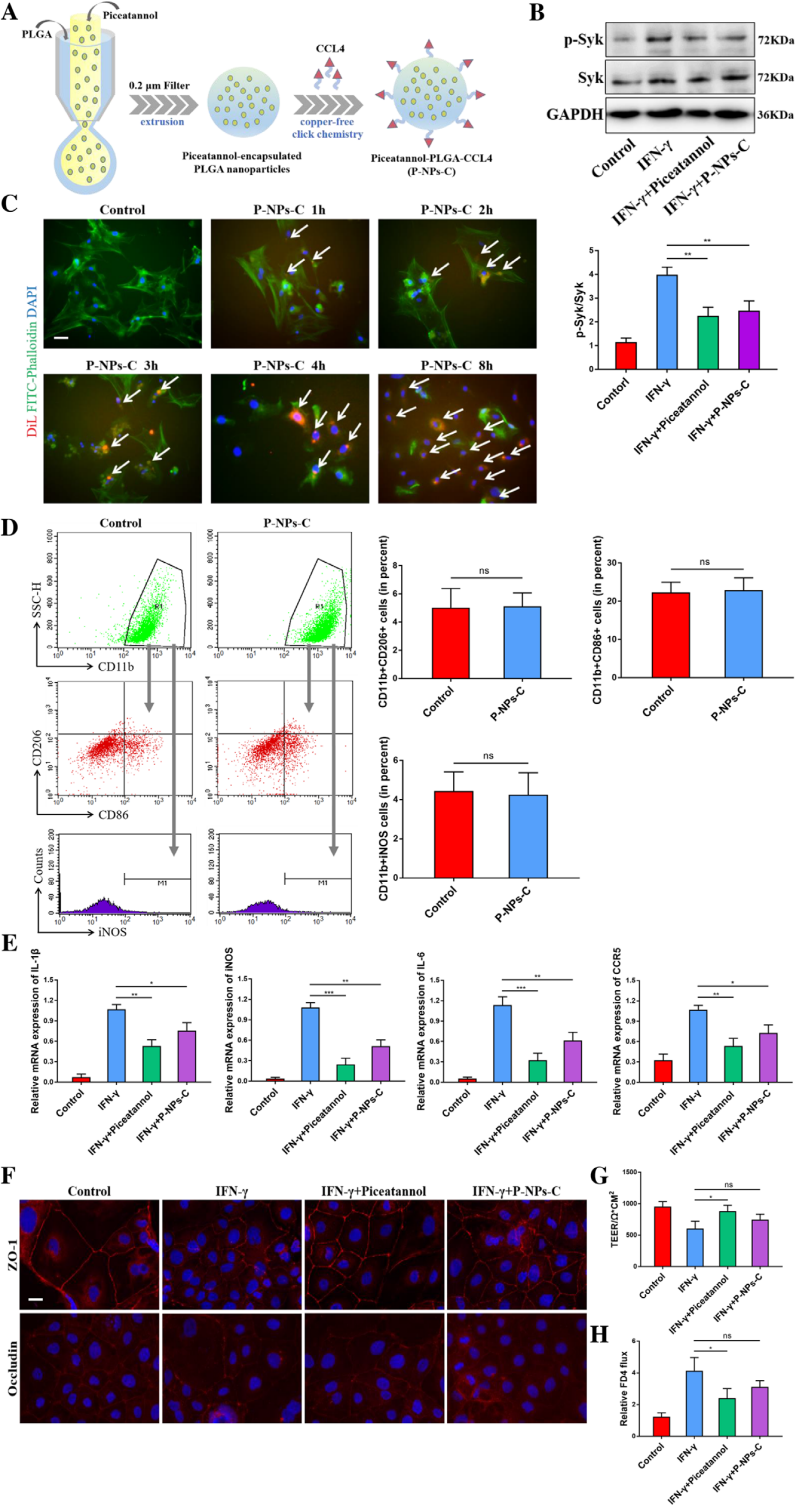
In vitro cellular uptake and therapeutic effect of P‐NPs‐C. (A) A double‐emulsion microfluidic system combined with copper‐free click chemistry technique was employed to prepare P‐NPs‐C. (B) P‐NPs‐C effectively inhibited IFN‐γ‐induced Syk phosphorylation in BMDMs. (C) Fluorescence images of cell internalization of P‐NPs‐C in BMDMs at different time points (1, 2, 3, 4, and 8 h). Cells in the absence of nanoparticles were set as a control. Scale bar = 20 μm. (D) Effect of P‐NPs‐C on the polarization of BMDMs. (E) Inhibition of inflammatory markers by P‐NPs‐C in BMDMs. Effect of P‐NPs‐C on intestinal epithelial barrier function (F) and tight junction proteins ZO‐1 and Occludin. Scale bar = 10 μm. (G) TEER and (H) FD4 flux in Caco‐2 cells. Data were shown as mean values ± SD. *P < 0.05, **P < 0.01, ***P < 0.001, and ns, no significance

Due to the poor bioavailability and pharmacokinetics of small‐molecule tyrosine kinase inhibitors, we prepared Syk inhibitor‐encapsulated PLGA nanoparticles to improve the bioavailability and therapeutic efficacy of piceatannol. Furthermore, to increase the specificity and to reduce the potential adverse effects, PLGA nanoparticles containing Syk inhibitor were conjugated to CCL4 (P‐NPs‐C) to deliver Syk inhibitor directly to CCR5‐expressing macrophages. CCR5 expression levels were much higher in IBD patients than healthy control, and almost all CCR5 protein were localized on F4/80+ macrophages (Figure 1C). P‐NPs‐C were prepared using a double‐emulsion microfluidic system combined with copper‐free click chemistry technique, which could improve the bioavailability and therapeutic efficacy of piceatannol (Figure 3A). As shown in representative SEM image, P‐NPs‐C were spherical in shape and exhibited uniform size distributions (Figure S1A). In addition, a dynamic light scattering and a Nano ZS Zetasizer were employed to detect the particle size and zeta potential of P‐NPs‐C. P‐NPs‐C had average diameter of 238.7 ± 2.4 nm and zeta potential of −26.43 ± 1.28 mV (Figures S1B and S1C). The physical stability of the P‐NPs‐C was observed and the particle diameter and zeta potential remained relatively stable after 30 days (Figures S1D and S1E). Next, we examined the release profiles of piceatannol from P‐NPs‐C in vitro and found that P‐NPs‐C achieved cumulative piceatannol releases of about 40% at the first 8 h and about 60% over 48 h (Figure S1F). Moreover, the drug uptake assay showed that there was a more uptake of piceatannol in free form (about 50% at 48 h) compared with P‐NPs‐C (about 42% at 48 h) in BMDMs (Figure S1G).

### 3.4 Biocompatibility of P‐NPs‐C in vitro and in vivo

Biocompatibility is an essential issue that should be considered for such nanoparticle carrier systems. To assess the cell viability and proliferation, we studied the effects of P‐NPs‐C on BMDMs in vitro using CCK‐8 assay. We found that treatment with piceatannol or P‐NPs‐C for 24 h did not influence the viability of BMDMs (Figure S1H). To evaluate potential toxicity of P‐NPs‐C in vivo, the normal mice were orally administered with P‐NPs‐C or PBS control for 7 days. After 7 days of administration, body weight was compared; blood was collected for hematological analysis; colonic mucosa was collected for leukocyte infiltration detection; and tissues from different parts of gastrointestinal tract and major organs were extracted for histological analysis. Compared with the control group, there were no notable difference in body weight and hematological parameters in the P‐NPs‐C treatment group (Figures S2A and S2B). There was no difference in CD45+ leukocyte infiltration of colonic mucosa between the two groups (Figure S2C). Additionally, no remarkable sign of toxicity was observed in the entire gastrointestinal tract (Figure S2D) and main organs (Figure S2E). These results indicated that P‐NPs‐C exhibited outstanding biocompatibility both in vivo and in vitro and can be used as a safe drug delivery carrier.

### 3.5 P‐NPs‐C inhibited Syk signal and its effects in vitro

Following successful synthesis of P‐NPs‐C, we evaluated the efficacy of these nanoparticles in vitro. As shown in Figure 3B, P‐NPs‐C presented a comparable effect to free piceatannol, both of which could inhibit IFN‐γ‐induced Syk phosphorylation in BMDMs. To verify the targeting ability of P‐NPs‐C, in vitro cellular uptake tests using DiL‐labeled P‐NPs‐C in BMDMs were performed. A weak intracellular DiL red fluorescence after 1 h of incubation was observed and the fluorescence density gradually increased with the increase of time, and much stronger fluorescence signal was presented in cytoplasm or on the membrane of BMDMs after 8 h of incubation (Figure 3C). CCL4, also known as macrophage inflammatory protein 1β (MIP1β), is involved in inflammatory response after binding with CCR5 receptor on macrophage. Therefore, in order to exclude the proinflammatory effect of nanoparticles themselves, we measured the polarization of BMDMs after adding P‐NPs‐C. Flow cytometry results of BMDMs showed that P‐NPs‐C did not boost the polarization of proinflammatory macrophages (Figure 3D). We further studied the therapeutic effects of P‐NPs‐C in IFN‐γ‐stimulated BMDMs and found that both piceatannol and P‐NPs‐C significantly inhibited the inflammatory gene expression (Figure 3E). Intriguingly, we observed that piceatannol was more effective than P‐NPs‐C, which is most likely owing to the slow and sustained drug release properties of PLGA nanoparticles.^23^ In addition, for the purpose of translation of these findings, we also observed the effects of P‐NPs‐C on human monocyte THP‐1 cell line. Consistent with the results in BMDMs, we observed that both piceatannol and P‐NPs‐C could inhibit IFN‐γ‐stimulated Syk phosphorylation and inflammatory genes expression in THP‐1 cell, whereas the empty NPs could not (Figure S2). Next, we evaluated the effect of P‐NPs‐C on intestinal epithelial barrier. As shown in Figure 3F, both piceatannol and P‐NPs‐C could restore Syk activation‐caused disruption of tight junction protein. Notably, although P‐NPs‐C could increase TEER and decrease FD4 flux of Caco‐2 monolayer barrier, it was not statistically different (Figures 3G and 3H). Taken together, these results supported that P‐NPs‐C could be efficiently internalized by macrophages and presented a comparable therapeutic efficacy to free piceatannol in vitro, which further suggested that slow and sustained release of piceatannol would be desirable for in vivo experiment.

### 3.6 Targeted uptake of P‐NPs‐C by the colonic mucosa and amelioration signs of experimental colitis

**FIGURE 4:**
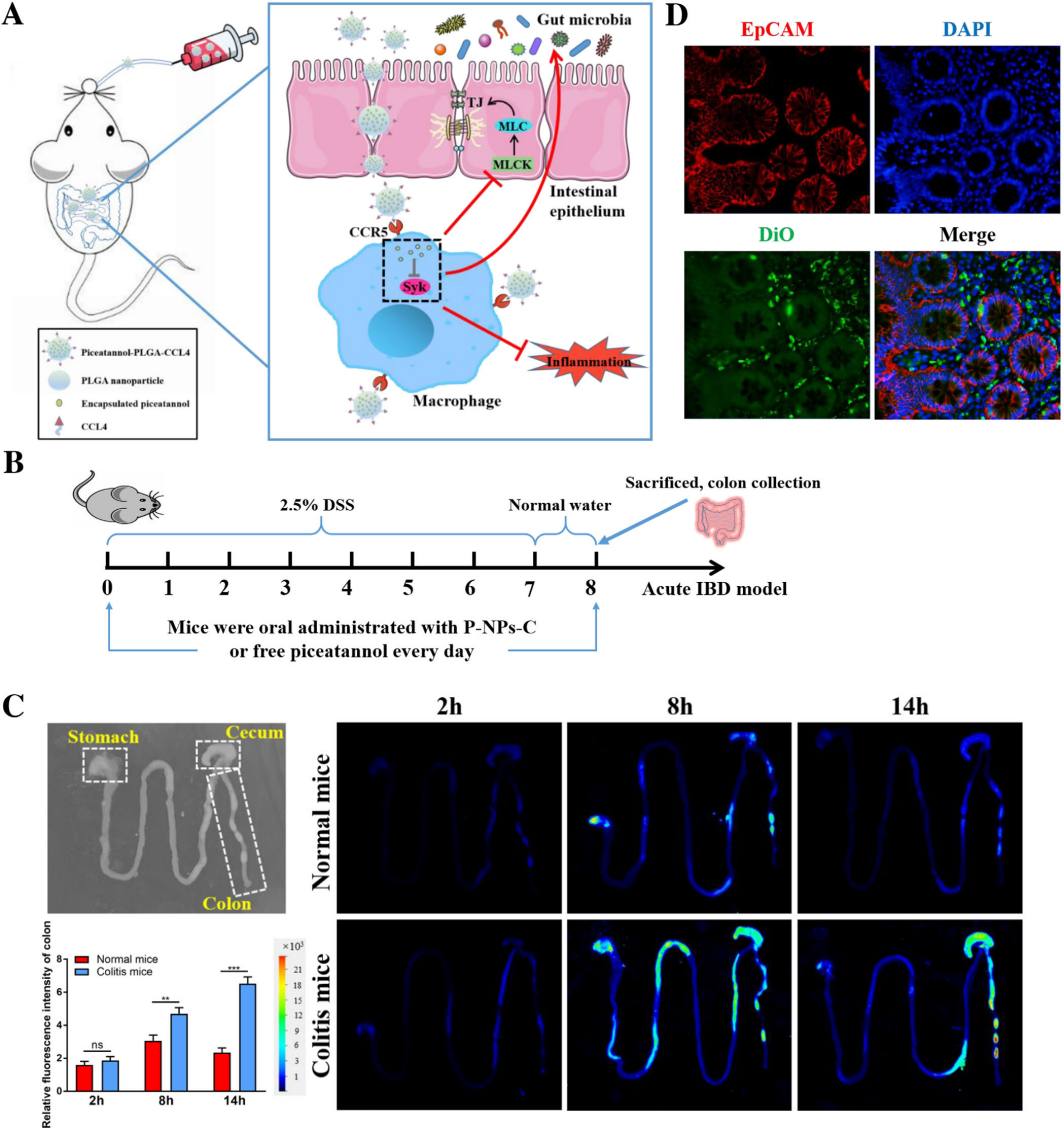
In vivo biodistribution of P‐NPs‐C. (A) Schematic illustration of therapeutic effect of P‐NPs‐C. Oral administration of P‐NPs‐C can target immune cells in lamina propria of inflamed colon mucosa through CCR5 receptor, thus inhibiting intestinal inflammation, restoring the intestinal barrier function, and improving intestinal microflora dysbiosis. (B) Illustration of the experimental design. Mice were provided with 2.5% DSS for 7 days with simultaneous daily oral administration of P‐NPs‐C or piceatannol (*n* = 8–12/group). (C) Bright field image of gastrointestinal tract from normal mice (without P‐NPs‐C treatment) and fluorescence images of gastrointestinal tract from normal mice and colitis mice. Typical images of gastrointestinal tract showing bio‐distribution of orally administered P‐NPs‐C at different time points (2, 8, and 14 h). (D) Frozen sections of colitis tissues after drug administration. Red, epithelium (EpCAM antibody); green, DiO; blue, DAPI (nucleus)

We further investigated whether P‐NPs‐C could achieve enhanced accumulation in inflamed colon when orally administered to mice (Figure 4A). Normal and colitis mice were orally gavaged with DiR‐loaded P‐NPs‐C within the hydrogel system, and an infrared imaging system was used to detect the DiR fluorescent dye in gastrointestinal tract after 2‐, 8‐, and 14‐h administration. We observed that colitis mice treated with P‐NPs‐C /DiR presented much stronger fluorescent intensity in the colon compared with normal mice after both 8‐ and 14‐h administration (Figure 4C). We also evaluated the biodistribution and localization of P‐NPs‐C in other main organs, including heart, liver, spleen, lung, and kidney. As shown in Figure S3, the DiR fluorescence intensity in liver, spleen, and kidney of colitis mice was much stronger than that of normal group. Additionally, the fluorescence signal in liver was dramatically higher than that in other organs, illustrating that P‐NPs‐C were excreted through liver metabolism. Fluorescence staining of colon tissues from colitis mice verified that oral administration of P‐NPs‐C/DiO led to the delivery of P‐NPs‐C to colon and their uptake by immune cells in colonic lamina propria (Figure 4D). Further, fluorescence staining of F4/80 confirmed that most of these immune cells taking in P‐NPs‐C were macrophages (Figure S4D).

**FIGURE 5:**
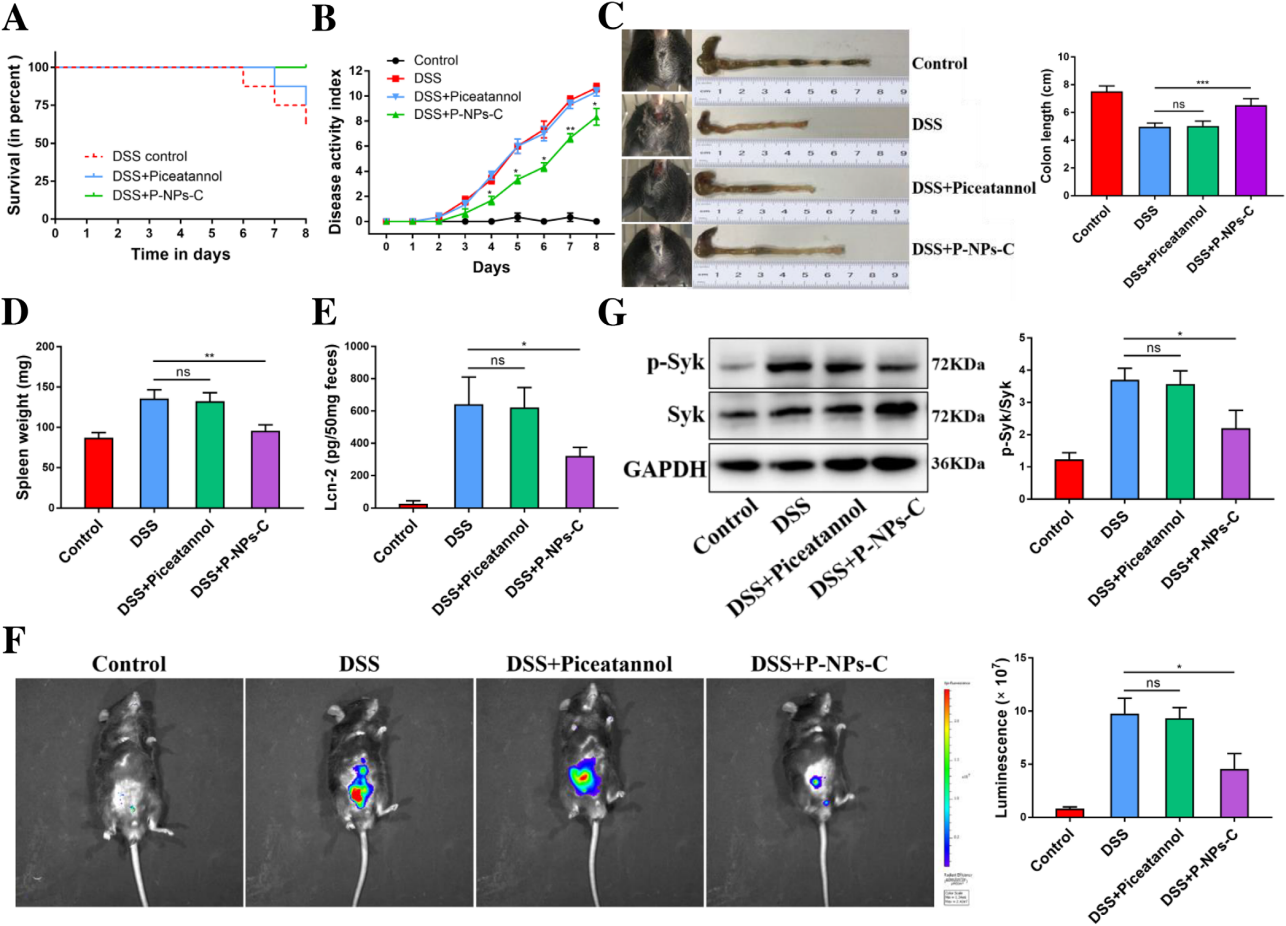
Therapeutic outcomes of P‐NPs‐C. (A) Survival rate of colitis mice orally received P‐NPs‐C or free piceatannol. (B) Disease activity index (DAI) of colitis mice orally received P‐NPs‐C or free piceatannol. (C) Photographs of rectal bleeding and colon length, (D) spleen weight, and (E) fecal Lcn‐2 level. (F) Bioluminescence images of mice treated with an inflammation probe (XenoLightTM; MA, USA), as captured by IVIS. At 5 min postinjection, images were obtained using a 5‐min exposure time. (G) P‐NPs‐C effectively inhibited Syk phosphorylation in experimental colitis. Data were shown as mean values ± SD. *P < 0.05, **P < 0.01, ***P < 0.001, and ns, no significance

We next assessed whether P‐NPs‐C could improve signs of disease. Mice were supplied with 2.5% DSS for 7 days with simultaneous daily gavage of P‐NPs‐C or piceatannol (Figure 4B). Strikingly, after one cycle of DSS, DSS plus P‐NPs‐C group showed a significant increased survival rate compared with DSS control and DSS plus piceatannol groups (Figure 5A). In line with this observation, decreased DAI score, reduced rectal bleeding, and increased colon length that coincided with decreased spleen weight were exhibited in DSS plus P‐NPs‐C group (Figure 5B–D). The fecal Lcn‐2 level on Day 8 in DSS plus P‐NPs‐C group was dramatically lower than that in DSS control and DSS plus piceatannol groups (Figure 5E). To further monitor the treatment effects in colitis mice, we employed in vivo bioluminescence imaging with the inflammation probe. As shown in Figure 5F, DSS and DSS plus piceatannol groups showed strong fluorescent signal, which reflected serious luminal inflammation. In contrast, this fluorescent signal was much reduced in DSS plus P‐NPs‐C‐treated mice. Moreover, we evaluated the inhibition efficacy of P‐NPs‐C in vivo and found that P‐NPs‐C could significantly inhibit DSS‐induced Syk phosphorylation, whereas oral administration of piceatannol had little influence (Figure 5G). Taken together, these data indicated that P‐NPs‐C exhibited excellent targeted uptake by inflamed colon and thus improved disease signs of experimental colitis.

### 3.7 Oral administration of P‐NPs‐C alleviated intestinal inflammation and improved intestinal barrier dysfunction during colitis

**FIGURE 6:**
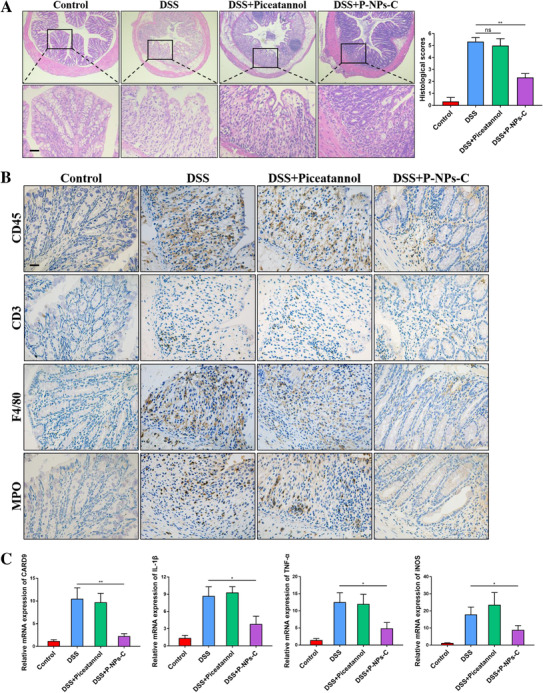
P‐NPs‐C alleviated intestinal inflammation during colitis. (A) Mucosal inflammation analyzed by histopathology was displayed (*n* = 6–10/group). Scale bars = 100 μm. (B) Cellular fractions of CD45^+^ leukocyte, CD3^+^ T cells, F4/80^+^ macrophages, and MPO^+^ neutrophils in colonic mucosa of DSS‐treated mice that were orally administered with P‐NPs‐C or piceatannol were determined by immunohistochemistry staining. Representative images were displayed. Scale bars = 100 μm. (C) The mRNA expressions of various inflammatory genes were quantified by real‐time PCR. Data were shown as mean values ± SD. *P < 0.05, **P < 0.01, and ns, no significance

To assess the effects of P‐NPs‐C on the amelioration of intestinal inflammation, histopathological staining of colon tissues from different treatment groups was performed. Compared with DSS control and DSS plus piceatannol groups, DSS plus P‐NPs‐C group exhibited a restoration of mucosal structure and less intestinal inflammation (Figure 6A). To further study the grade of intestinal inflammation, we next observed the immune cells infiltration in the colonic lamina propria by immunohistochemistry staining. Corresponding to the decreased disease signs, less CD45+ leukocytes infiltration was found in DSS plus P‐NPs‐C group (Figure 6B). Compared with DSS control and DSS plus piceatannol groups, the fractions of CD3+ T cells, F4/80+ macrophages, and MPO+ neutrophils were also decreased in DSS plus P‐NPs‐C group (Figure 6B). We also determined the mRNA levels of proinflammatory genes in these groups. As shown in Figure 6C, the expression levels of the proinflammatory genes, CARD9, IL‐1β, TNF‐α, and iNOS, were markedly reduced in colon tissues of DSS plus P‐NPs‐C group.

**FIGURE 7:**
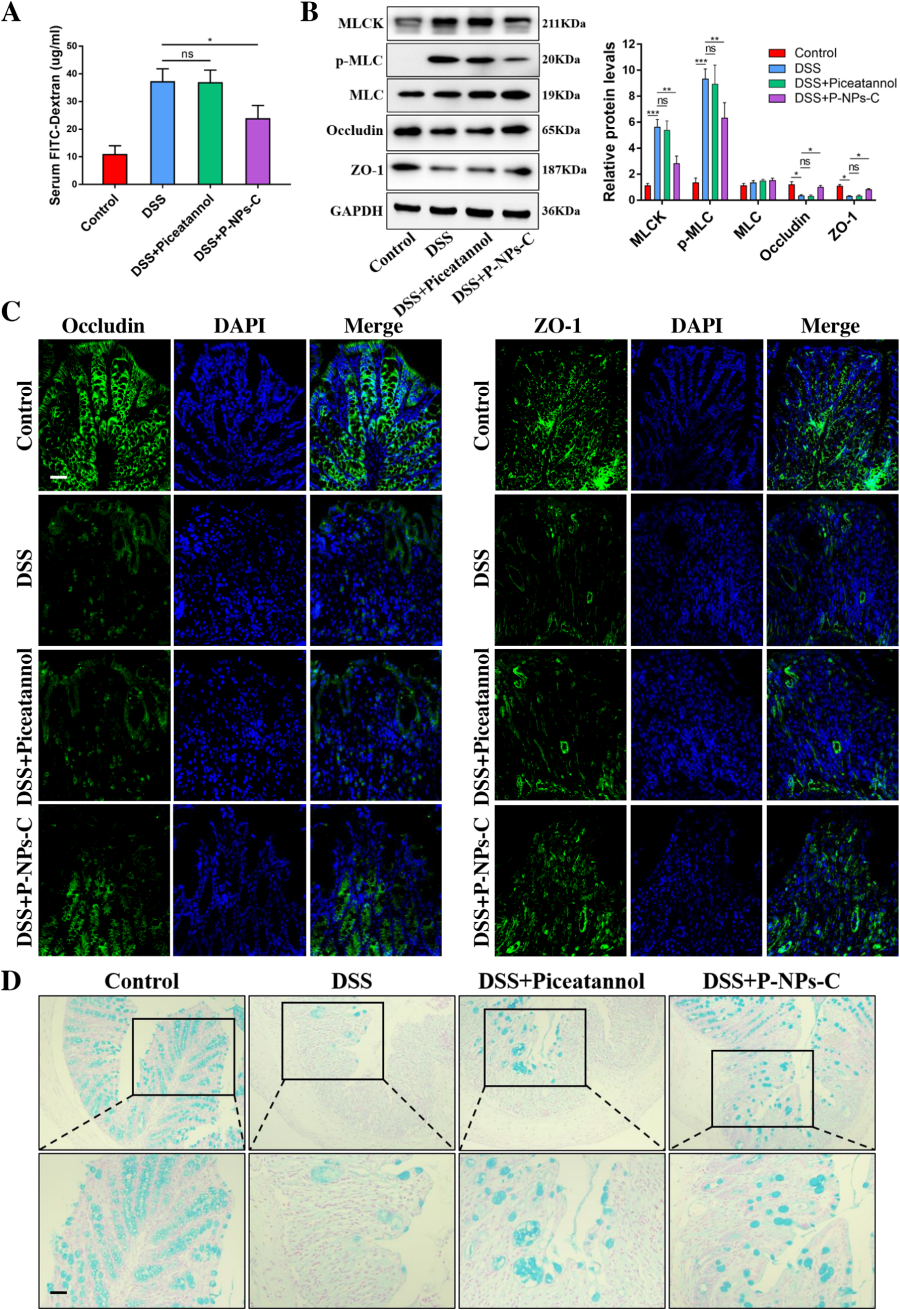
P‐NPs‐C improved intestinal barrier dysfunction during colitis. (A) Serum concentration of FITC–Dextran in colitis mice treated with P‐NPs‐C or free piceatannol (*n* = 5–10/group). (B) Oral administration of P‐NPs‐C downregulated the protein levels of MLCK and the phosphorylation of MLC, and restored Occludin and ZO‐1 during colitis. (C) The expression of Occludin and ZO‐1 was determined by immunofluorescence. (D) Alcian blue staining of colon tissues. Scale bar = 50 μm. Data were shown as mean values ± SD. *P < 0.05, **P < 0.01, ***P < 0.001, and ns, no significance

We next investigated the effect of P‐NPs‐C on intestinal barrier function. As shown in Figure 7A, the serum concentration of FITC–Dextran, a permeability tracer, was reduced a lot in colitis mice treated with P‐NPs‐C. The proteins expression of MLCK/p‐MLC pathway was decreased and tight junction proteins Occludin and ZO‐1 were restored in DSS plus P‐NPs‐C group (Figure 7B). Fluorescence staining of Occludin and ZO‐1 confirmed that oral administration of P‐NPs‐C promoted the recovery of tight junction proteins (Figure 7C). Alcian blue staining indicated a restoration of mucous cells in colon tissues in DSS plus P‐NPs‐C group compared with DSS control and DSS plus piceatannol groups (Figure 7D). These results demonstrated that P‐NPs‐C showed an excellent anti‐inflammatory therapeutic efficacy in vivo and improved intestinal barrier function by modulating MLCK/p‐MLC pathway.

### 3.8 Impact of P‐NPs‐C on intestinal microbiota

**FIGURE 8:**
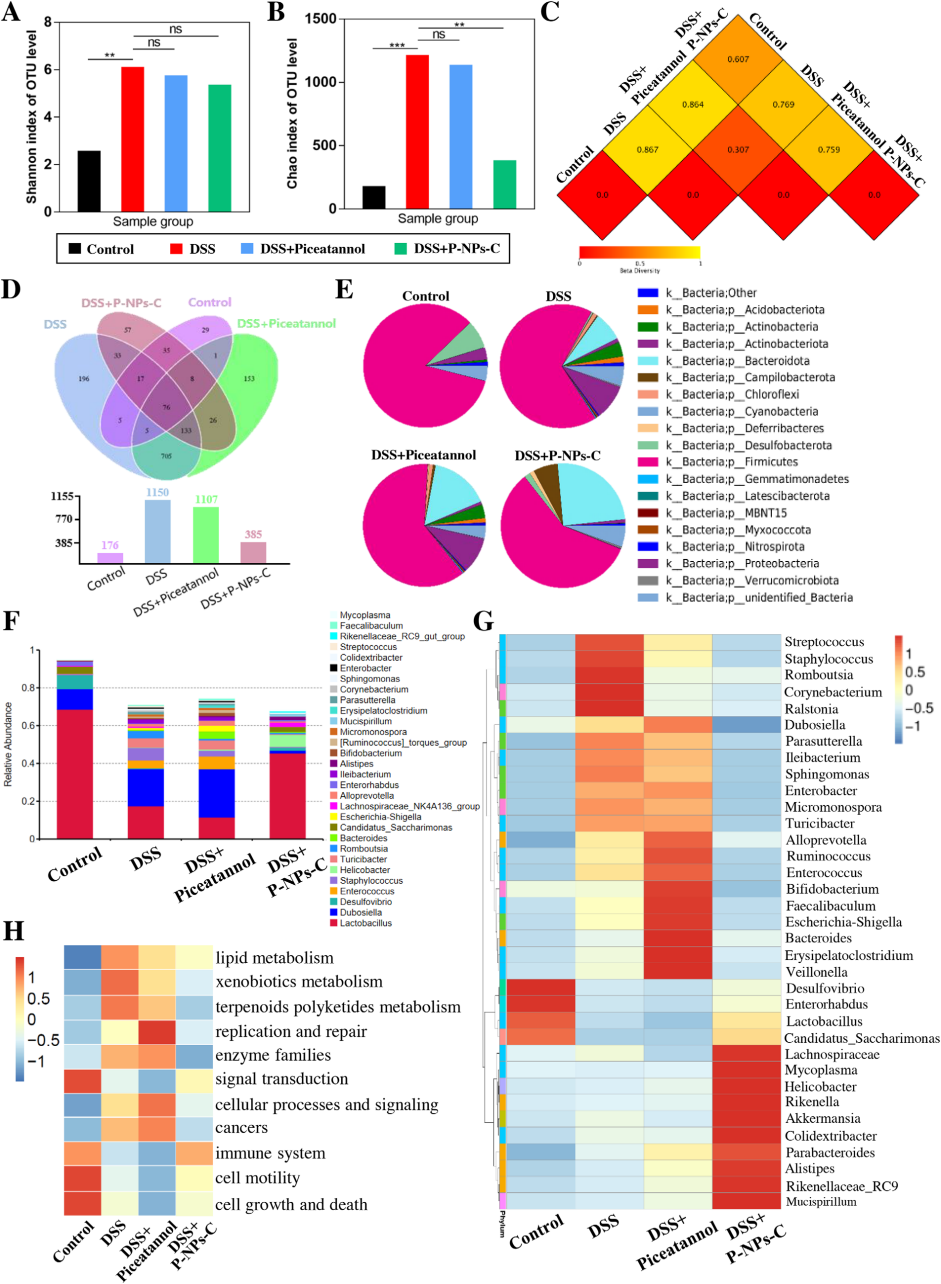
Evaluation of remodeling effect of P‐NPs‐C on intestinal microbiota. (A) Shannon and (B) Chao index of operational taxonomic unit level of intestinal flora. (C) UniFrac distances analysis and (D) Venn diagram of shared and unique bacterial species of mice in different groups. (E) The composition of dominant bacteria (Phylum) in the feces of mice. (F) Colony composition histogram illustrating relative taxonomic composition of intestinal bacteria at the genus level in different groups. (G) Heatmap of the composition of gut microbiota in different groups. (H) Predicted functional changes with obvious difference

Intestinal microbiota dysbiosis plays an essential role during the pathogenesis of IBD,^24^ and accumulating evidence has demonstrated that activation of Syk signal is largely attributed to abnormal intestinal microbiota.^25^, ^26^, ^27^ To evaluate whether P‐NPs‐C was able to improve intestinal microbiota dysbiosis, 16S rRNA sequencing was performed to explore intestinal microbiome in feces. As shown in Figures S5A and S5B, the richness and evenness of fecal samples were appropriate. Treatment of DSS, DSS plus piceatannol, and DSS plus P‐NPs‐C resulted in marked microbiota number and abundance changes in feces at the level of phylum, class, order, family, genus, and species (Figures S5C and S5D). DSS‐treated group showed a high species diversity (Shannon index) of gut microbiota, whereas DSS plus piceatannol and DSS plus P‐NPs‐C groups did not show species diversity compared with DSS control group (Figure 8A). Meanwhile, DSS‐treated group also presented a higher community richness, and mice received P‐NPs‐C were similar to the healthy control group and showed significant differences compared to the DSS group (Figure 8B). No apparent variation was observed in community abundance between the DSS and DSS plus piceatannol groups. The UniFrac distances analysis representing beta diversity showed that there were an obvious community difference between control and DSS groups, whereas this difference was reduced between DSS plus P‐NPs‐C and control groups (Figure 8C). In addition, the species Venn diagram analysis was performed to show the overlaps among the four groups. There were significant species differences among the four groups and 76 Operational Taxonomic Units (OTUs) overlapped among all groups (Figure 8D), which demonstrated that existence of a certain type of bacterial type played a significant role in maintaining intestinal homeostasis.

The results presented in Figures 8E and S5E showed that the dominant bacteria in the healthy control group were *Firmicutes*, *Desulfobacterota*, *Proteobacteria*, and *Actinobacteriota*. In the DSS group, we found a rapid decrease in *Firmicutes*, *Desulfobacterota*, and *Actinobacteriota*, and a significant increase in *Proteobacteria* and *Bacteroidota*. DSS plus piceatannol group presented a similar phylum trend compared with DSS control group, whereas *Firmicutes*, *Desulfobacterota*, and *Actinobacteriota* increased and *Proteobacteria* reduced in the feces of mice treated with P‐NPs‐c. As shown in Figure S6A–C, comparative analysis of the class, order, and family‐level compositions showed that DSS insult significantly altered the abundance of intestinal flora, and P‐NPs‐C treatment restored these differences to a certain extent. Notably, as shown in Figures 8F and S6D, DSS group showed a unique dominant species composition compared to the healthy group at the genus or species level, that is, a rapid fall in beneficial symbiotic *Lactobacillus* and the harmful and pathogenic bacteria, including *Dubosiella*, *Enterococcus*, and *Staphylococcus*. The beneficial commensal *Lactobacillus* in the dominant flora raised, whereas the harmful and pathogenic bacteria reduced when mice were treated with P‐NPs‐C. Furthermore, the heat maps of compositions comparative analysis at phylum, class, order, family level (Figure S7) or genus level (Figure 8G) indicated that the gut microbiota compositions in DSS plus P‐NPs‐C group were significantly different from DSS control and DSS plus piceatannol groups, but resembled that in the healthy control group to a certain extent.

To further evaluate the differences among the four groups, the cylindrical coordinate analysis (PCoA) was conducted. The distances between P‐NPs‐C‐treated group and the healthy group showed certain difference at two‐dimensional PCoA axis, which reflected the therapeutic effects of P‐NPs‐C (Figure S6E). At different levels of phylogeny, key phylogenetic types were identified as distinguishable biomarkers. As shown in Figure 8, *Lactobacillus* was the most significant decreased bacteria and *Dubosiella* was the most significant increased bacteria in the DSS group at the phylum level. This phenomenon was rescued after P‐NPs‐C administration. Moreover, functional prediction analysis based on the differential OTUs revealed a notable enrichment for several biological processes including metabolism (e.g., carbohydrate, amino acid, and energy metabolism), genetic information processing (e.g., replication and repair and translation), and environmental information processing (e.g., membrane transport and signal transduction) (Figures S6F and S6G). Markedly, comparison between DSS group and healthy control group indicated that some functions differed obviously after DSS insult including enhanced lipid metabolism, xenobiotics metabolism, enzyme family, and risk of cancers and reduced immune function, cell motility, and signal transduction (Figure 8H). Administration of P‐NPs‐C significantly ameliorated these functional changes (Figure 8H). Collectively, these results demonstrated that disruption of intestinal microbiota homeostasis would result in inflammation or other diseases, and P‐NPs‐C promoted the remodeling of imbalanced microbiota toward a healthy flora.

## 4 DISCUSSION

The medication for IBD therapy over the last two decades has gradually transitioned from reliance on aminosalicylates, corticosteroids, and immunomodulators to biological therapies based on monoclonal antibodies.^28^ Nevertheless, there is still a large unmet need for novel therapeutic approaches as many patients do not have positive response to clinically approved drugs, such as TNF‐α antibody, ustekinumab, and vedolizumab.^29^, ^30^, ^31^ The development of targeted oral delivery systems provides a strong research potential for diseases affecting colon, notably for IBD. In recent years, nanotechnology has emerged as a novel and efficient method for targeted drug delivery to specific sites of inflamed colon. Upon oral administration, nanoparticle‐based drug carrier can provide high local concentration of drug at the site of lesion and reduced drug degradation, which promotes prolongation of pharmacological activities and drug efficacy, and has the potential to decrease systemic adverse effects because it does not affect normal tissues.^32^ The evolving nanoparticle‐based drug carrier systems have been shown to be ideally suitable for oral administration of colon‐targeted drugs.^33^ These carrier systems are able to encapsulate or dissolve the objective drug therapeutically and then directly deliver the drug to the inflammatory site after breaking through physiological barriers. In some cases, active drugs may be attached to or adsorbed on the nanoparticles to achieve satisfied therapeutic effect. Currently, the commonly used nanoparticle‐based drug delivery systems include pH‐dependent, reactive oxygen species‐responsive, hydrogel‐based, and active targeting‐dependent nanodelivery systems. Further, nanoparticles derived from natural sources, for example, extracellular vesicles, are considered as novel potential carrier systems due to their outstanding safety and effectiveness.^34^

In this study, we prepared the PLGA nanoparticles loaded with Syk inhibitor piceatannol and grafted the chemokine CCL4 onto the nanoparticles to enhance drug targeting. Due to the excellent properties of biocompatibility, biodegradability, ease of formulation, and tunable sustained drug release, PLGA has emerged as a promising carrier in a variety of biomedical applications including inflammation, cancer, vaccination, and other diseases.^35^ PLGA can be hydrolyzed to lactic acid and glycolic acid biologically in vivo, which will be endogenously metabolized via the Krebs cycle.^36^ Therefore, there is no systemic toxicity in drug delivery with PLGA. It is demonstrated that PLGA nanoparticles are internalized in cells through Clathrin‐mediated endocytosis and fluid‐phase pinocytosis.^37^ In in vitro experiments, we found that nanoparticle‐loaded piceatannol seems to be less effective than free form of piceatannol, which is actually due to the release kinetics of PLGA drug carrier. PLGA nanoparticles‐encapsulated drug is mainly degraded by hydrolysis after internalization. Penetration of water into polymer results in uniform bulk degradation of polymer matrix, which creates a passage for drug diffusion and release until complete polymer solubilization.^38^ That is to say, the physical and chemical characteristics of PLGA endow it with sustained‐release function as the drug carrier. However, an obvious disadvantage of PLGA nanoparticles is the extremely limited number of surface‐displayed functional groups available for targeting ligands.^39^ Certain cell receptors or transporters, such as CD44, folate receptor, and CCR5, are overexpressed during the inflammatory process, which provide great potential for active drug targeting.^20^ In the present study, we conjugated chemokine CCL4 on the piceatannol‐encapsulated PLGA nanoparticles, which can bind to its receptor CCR5 on the surface of inflammatory macrophages, greatly increasing the targeting ability of drugs.

Piceatannol, a natural hydroxylated stilbene analog and metabolite of resveratrol, has been demonstrated to exhibit anti‐inflammatory, antioxidative, antiaging, antiobesity, and anticancer effects similar to resveratrol, but has better bioavailability and stability than resveratrol.^40^ Piceatannol is mainly generated during the process of fruit ripening and microbial β‐glucosidases‐triggered fermentation.^41^ There has been accumulating evidence to reveal a crucial role of piceatannol in participating multiple physiological and pathophysiological by modulation of a range of signaling pathways.^42^ Piceatannol has been reported to inhibit oxidative stress by activating the PI3K/AKT, PTEN (phosphatase and tensin homolog)/AKT, MAPK cascade, and Sirt1 (sirtuin1)/FoxO1 signaling and alleviate inflammation by modulating NF‐κB and Nrf2/HO‑1 pathways.^42^, ^43^ In addition, piceatannol, as a selective inhibitor of Syk, can mitigate the disease state by directly inhibiting the pathological effect of Syk.

Notably, there is emerging data from both experimental and clinical studies implicating that Syk takes part in the pathogenesis of gut disorders, especially IBD. Mechanistically, our previous studies found that macrophage‐inducible C‐type lectin (Mincle) over‐activates Syk by sensing spliceosome‐associated protein 130 (SAP130) released from damaged intestinal epithelial cells during intestinal inflammation, and increased Syk phosphorylation is able to induce the production of inflammatory cytokines through NOD‐like receptor family pyrin domain containing 3 (NLRP3) inflammasome‐mediated macrophage pyroptosis, thus regulating intestinal mucosal immunity and promoting the disease progression of IBD.^6^, ^12^ Indeed, a previous study showed that Helminth Heligmosomoides polygyrus bakeri infection down‐modulates Syk and Syk‐coupled Dectin‐1 expression in resident intestinal dendritic cells (DCs), which induces regulatory DCs that rescue colitis and inhibit intestinal Ag‐induced T cell responses.^10^ Syk phosphorylation activated by DNAX adaptor protein 12 (DAP12) initiates an inflammatory signaling cascade in IBD and acts as an essential mediator during inflammation‐driven immune dysfunction of experimental colitis.^11^ In addition, the use of Syk inhibitors, Cerdulatinib, Fostamatinib, and TOP1210, can ameliorate severity of experimental colitis by inhibiting release of proinflammatory cytokines and reducing mucosal damage.^10^, ^15^, ^16^ Remarkably, another Syk inhibitor R406 is able to completely reverse the infliximab‐mediated decline in IL‐12p40 as well as the augment in IL‐10 in inflammatory macrophages.^17^

In this study, we found that Syk signaling not only promotes intestinal inflammation in IBD, but also destroys the intestinal epithelial barrier and causes intestinal microbiota dysbiosis. It is most probably because activation of Syk in macrophages produces some inflammatory mediators, which can activate the MLCK/p‐MLC pathway in intestinal epithelial cells, resulting in the remodeling of epithelial tight junction proteins and damage of intestinal barrier. Additionally, intestinal microbiota is essential to maintain the physiological function of gastrointestinal tract, and most of intestinal microbiome is anaerobic bacteria that reside in colon. These anaerobic bacteria produce a variety of hydrolytic and digestive enzymes when they recognize the substrates entering the colon.^44^ The biodegradable polymers utilized in nanoparticle‐based drug delivery carrier can be consumed as a source of carbon to be degraded into saccharides by the colonic microbiome.^45^ Syk is a common downstream coupled protein to transduce cell membrane receptors signaling. Such receptors, for example, C‐type lectin receptors, can be activated by different cell wall components from aberrant translocation of the fungal or bacterial pathogen, which further activate Syk and amplify the inflammatory cascade.^46^, ^47^ Thereby, nanoparticle‐based drug delivery systems composed of biodegradable polymer and Syk inhibitor piceatannol can collaboratively lessen the pathological effect caused by intestinal microbiota dysbiosis, and further modulate intestinal homeostasis.

In summary, we confirmed schematically a novel role of Syk signaling contributed to IBD. Thereafter we used a double‐emulsion microfluidic system combined with copper‐free click chemistry technique to prepare Syk inhibitor piceatannol‐loaded P‐NPs‐C nanoparticles. Furthermore, we showed that oral administration of P‐NPs‐C in a hydrogel system could significantly ameliorate intestinal inflammation and improve intestinal barrier dysfunction and intestinal microflora dysbiosis during experimental colitis (Figure 4A). This nanoparticle delivery system represents a promising novel approach for IBD treatment.

## AUTHOR CONTRIBUTIONS

JR and WG designed experiments. WG, JY, TZ, PL, FZ, and JL performed experiments. WG, ZH, HR, GG, and GW analyzed the data. JR, XW, and YZ supervised the research. WG drafted the manuscript. JR, XW, and YZ revised the manuscript. All authors read and approved the final manuscript.

## CONFLICT OF INTEREST

The authors declare no conflict of interest.

## Supporting information



Supporting Information

## Data Availability

The data underlying this article are available in the article and in its Supporting Information.
