# Erratum to “CCL4‐mediated targeting of spleen tyrosine kinase (Syk) inhibitor using nanoparticles alleviates inflammatory bowel disease”

**DOI:** 10.1002/ctm2.70434

**Published:** 2025-08-17

1

Gong W, Yu J, Zheng T, et al. CCL4‐mediated targeting of spleen tyrosine kinase (Syk) inhibitor using nanoparticles alleviates inflammatory bowel disease. Clin Transl Med. 2021;11(2):e339.

In this article, we realised an incorrect staining image was used in the Control group in Figure  6B, and an incorrect immunofluorescence image was used for ZO‐1 staining in the DSS+Piceatannol group in Figure 7C after a careful reexamination of the manuscript. Upon reviewing the original data, we realised that the mistake was made inadvertently during manuscript preparation. We have now replaced the correct images of Figures 6 and 7, while the description of Figures 6 and 7 in the results section of the original version does not need to be changed. We confirm that the errors did not affect the results, interpretation and conclusion of the study.

**FIGURE 6:**
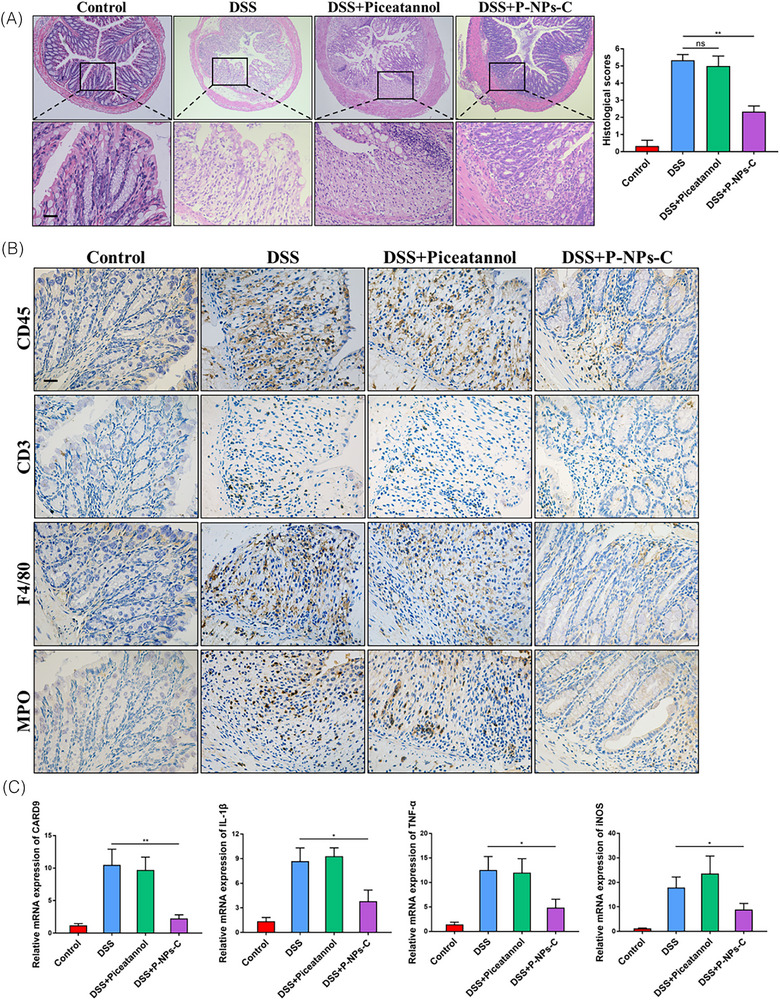
P‐NPs‐C alleviated intestinal inflammation during colitis. (A) Mucosal inflammation analyzed by histopathology was displayed (n = 610/group). Scale bars = 100 µm. (B) Cellular fractions of CD45+ leukocyte, CD3+ T cells, F4/80+ macrophages, and MPO+ neutrophils in colonic mucosa of DSS‐treated mice that were orally administered with P‐B;NPs‐C or piceatannol were determined by immunohistochemistry staining. Representative images were displayed. Scale bars + 100 µm. (C) The mRNA expressions of various inflammatory genes were quantified by real‐time PCR. Data were shown as mean values ± SD. *P < 0.05, **P < 0.01, and ns, no significance.

**FIGURE 7:**
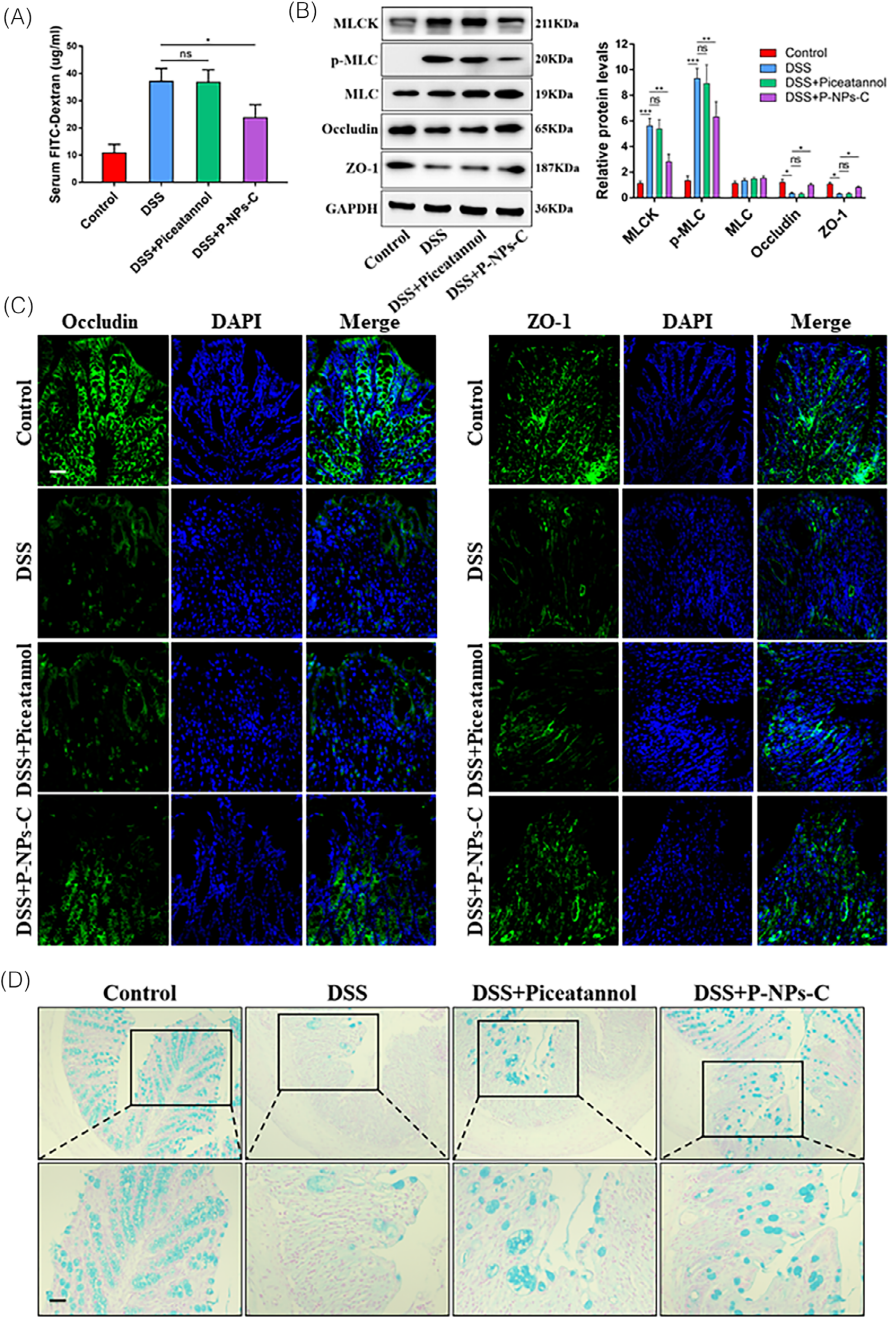


We apologise for this error.

