# Supplementary material for: CCL4‐mediated targeting of spleen tyrosine kinase (Syk) inhibitor using nanoparticles alleviates inflammatory bowel disease
Source: Clin Transl Med. 2021 Feb 17;11(2):e339. doi: 10.1002/ctm2.339 (PMC7888545; doi:10.1002/ctm2.339)
Supplement: Supplementary file 1 — Supporting Information [file CTM2-11-e339-s001.pdf]

Supplementary Materials for

CCL4 molecular conjugated of spleen tyrosine kinase (Syk) inhibition for alleviation of inflammatory bowel disease

Wenbin Gong[1,2], Jiafei Yu[2], Tao Zheng[2], Peizhao Liu[2], Fan Zhao[2], Juanhan Liu[2], Zhiwu Hong[2], Huajian Ren[2], Guosheng Gu[2], Gefei Wang[2], Xiuwen Wu[2]*, Yun Zhao[3]*, Jianan Ren[1,2]*

1. School of Medicine, Southeast University, Research Institute of General Surgery, Jinling Hospital, 210009 Nanjing, China
2. Research Institute of General Surgery, Jinling Hospital, 210002 Nanjing, China
3. Department of General Surgery, BenQ Medical Center, The Affiliated BenQ Hospital of Nanjing Medical University, Nanjing, P. R. China

Correspondence to:

*Xiuwen Wu, Email: lygwxw@163.com, *Yun Zhao, Email: zhaoyun056@gmail.com, and *Jianan Ren, Email: jiananr@gmail.com

Materials and Methods

Bone marrow derived macrophage preparation: Bone marrow-derived macrophages (BMDMs) were isolated from C57BL/6 mice. Briefly, Femur and tibia were flushed by a syringe. Bone marrow cells were resuspended and cultured in RPMI 1640 containing 10% FBS and 10 ng/ml M-CSF after excluding red blood cells. On the third day, 10 ng/ml M-CSF was added to the RPMI 1640 medium again. Cells were successfully differentiated into BMDMs on day 7. Phenotypic characterization of BMDMs were confirmed by flow cytometry.

Biocompatibility assays: To assess the effects of P-NPs-C on cell proliferation, the Cell Counting Kit-8 (CCK-8, Sigma-Aldrich) was used based on the manufacturer's protocol. Briefly, BMDMs were plated into 12-well plates and incubated with piceatannol (50 μM) or P-NPs-C for 48 h. CCK-8 solution was added to each well in the ratio of 1/10 and incubated for 1 h at 37 °C. Subsequently, 200 μL of reaction solution from each well was transferred to a new 48-well plate, and the absorbance at 450 nm wavelength was measured with a spectrophotometric microplate reader (Synergy HT, BioTek).

For the biocompatibility of P-NPs-C in vivo, mice were oral administrated with 20 mg of P-NPs-C dissolved in PBS or PBS control for 7 days. Body weight was recorded and blood, stomach, duodenum, jejunum, ileum, colon, colonic mucosa and main organs including heart, liver, spleen, lung and kidney were extracted. Blood cells

analyses were performed using a hematology analyzer (Nihon Kohden, Japan) and leukocyte infiltration of colonic mucosa were determined by flow cytometry.

TEER and paracellular FD-4 flux measurements: Caco-2 cells were seeded on the apical chamber of the 12-well transwell system and TEER was measured with the Millicell ERS-2 system (Millipore, Billerica, MA, USA). TEER values were corrected for background resistance due to filter and fluid resistance, and calculated as $\Omega \cdot cm^2$. Paracellular permeability of Caco-2 monolayer was analyzed by paracellular FD-4 flux (Sigma-Aldrich). Caco-2 cells were grown on transwell filters into monolayers, then rinsed with PBS and incubated in Hank's balanced salt solution for 2 hours supplemented with 1 mg/mL FD-4 solution. Fluorescence intensity of 100 μl supernatant from basolateral chamber was detected using a Synergy H2 microplate reader (BioTek Instruments, Winooski, VT, USA).

Quantitative real-time PCR: The mRNA levels of tissues and cells were detected by quantitative real-time PCR. Briefly, total RNA was extracted with a TRIzol reagent (Invitrogen, Carlsbad, CA, USA) and reverse-transcribed with M-MLV Reverse Transcriptase kit (ELK Biotechnology, EQ002, Wuhan, China). Afterwards, 1 μl of template was added in a 10-μl reaction containing 1.0 μl of each primer and 5 μl of QuFast SYBR Green PCR Master Mix (ELK Biotechnology, EQ001, Wuhan, China). Primers were showed in supplementary table S2. Results were normalized with GAPDH and shown as relative expression value.

Flow cytometry: The BMDMss were collected and washed twice with PBS. Antibodies for surface staining were as follows: CD11b (14-0112-82, eBioscience), CD206 (12-2061-82, eBioscience), CD86 (14-0862-82, eBioscience), iNOS (14-5920-82, eBioscience). Samples were incubated with the antibodies at 37 °C for 30 min, then run on an FACS Calibur (BD Bioscience) and analyzed by the FlowJo software (Treestar Inc).

Effects of P-NPs-C on intestinal microbiota: Mice were moved to sterilized environment and feces were collected before sacrifice. Total genome DNA from feces was extracted through CTAB/SDS method. 16S rRNA genes of different regions (16S V4/16S V3/16S V3-V4/16S V4-V5) were amplified with specific primer. PCR reactions were performed with 15 μL of Phusion® High-Fidelity PCR Master Mix (New England Biolabs), 10 ng template DNA and 0.2 μM of forward and reverse primers. Sequencing libraries were generated using the TruSeq® DNA PCR-Free Sample Preparation Kit (Illumina, USA) according to manufacturer's recommendations. The library was sequenced on an Illumina NovaSeq platform and 250 bp paired-end reads were generated. Sequences analysis were performed by Uparse software (Uparse v7.0.1001, http://drive5.com/uparse/) and Silva Database (http://www.arb-silva.de/) was used to annotate taxonomic information.

Supplementary Figure legends

Supplementary Figure 1. Characterization of P-NPs-C. SEM image (A), size distributions (B) and zeta potential (C) of P-NPs-C. Variations of hydrodynamic diameter (D) and zeta potential (E) of P-NPs-C during incubation for 30 days. F) Release profiles of piceatannol from P-NPs-C in medium as analyzed at different time intervals (0, 8, 16, 24, 32, 40 and 48 h). G) Drug uptake by BMDMs as measured at different time intervals (0, 8, 16, 24, 32, 40 and 48 h). H) Biocompatibility of P-NPs-C of on BMDMs in vitro using CCK-8 assay. Scale bars=200 nm. Data were shown as mean values ± SD. * < 0.05.

Supplementary Figure 2. Therapeutic effect of P-NPs-C on human monocyte THP-1 cell line. A) P-NPs-C effectively inhibited IFN-γ-induced Syk phosphorylation in THP-1 cells. B) Inhibition of inflammatory markers IL-1β, IL-6 and iNOS by P-NPs-C in THP-1 cells. Data were shown as mean values ± SD. * < 0.05, ** < 0.01, *** < 0.001, ns, no significance.

Supplementary Figure 3. Biocompatibility of P-NPs-C in vivo. Mice were oral administrated with P-NPs-C (20 mg in PBS) or PBS (control) for 7 days (n=6/group). A) Body weight change as measured in each group and normalized by the corresponding initial body weight. B) Blood was collected by intracardiac puncture with a syringe and hematological analyses were performed using an automatic hematology analyzer. C) Leukocyte infiltration of colonic mucosa between the two groups determined by flow cytometry. Toxicity of P-NPs-C toward gastrointestinal tissues (stomach, duodenum, jejunum, ileum, and colon) (D) and main organs (heart, liver, spleen, lung, and kidney) (E) evaluated by histological staining. Scale bars=100 μm. Data were shown as mean values ± SD.

Supplementary Figure 4. Biodistribution and localization of P-NPs-C in other main organs. A) Bright field image of heart, liver, spleen, lung and kidney from normal

mice (without P-NPs-C treatment). B) Fluorescence images of heart, liver, spleen, lung and kidney from normal mice and colitis mice. Typical images of gastrointestinal tract showing bio-distribution of orally administered P-NPs-C at 8h and 14h. C) Relative fluorescence intensity of main organs at 14h. D) Frozen sections of colitis tissues after drug administration. Red, macrophage (F4/80 antibody); green, DiO; blue, DAPI (nucleus).

Supplementary Figure 5. Impact of P-NPs-C on intestinal microbiota. Rank-abundance curve (A) and rarefaction curve (B) of different mice groups. C) Microbiota number changes in feces at the level of phylum, class, order, family, genus and species in different groups. D) Microbiota abundance changes in feces at the level of phylum, class, order, family, genus and species. E) Comparative analysis of species level of different groups.

Supplementary Figure 6. Comparative analysis of the phylum (A), class (B), order (C) and family (D)-level compositions of different groups. E) Principal coordinates analysis (PCoA) of gut microbiota in different groups. F, G) Functional prediction analysis revealed a significant enrichment for several biological processes including metabolism, genetic information processing and environmental information processing.

Supplementary Figure 7. Heatmaps of compositions comparative analysis at phylum, class, order, family levels of different groups.

Supplementary Figure 8. Evolutionary tree at different levels of phylogeny in different groups.

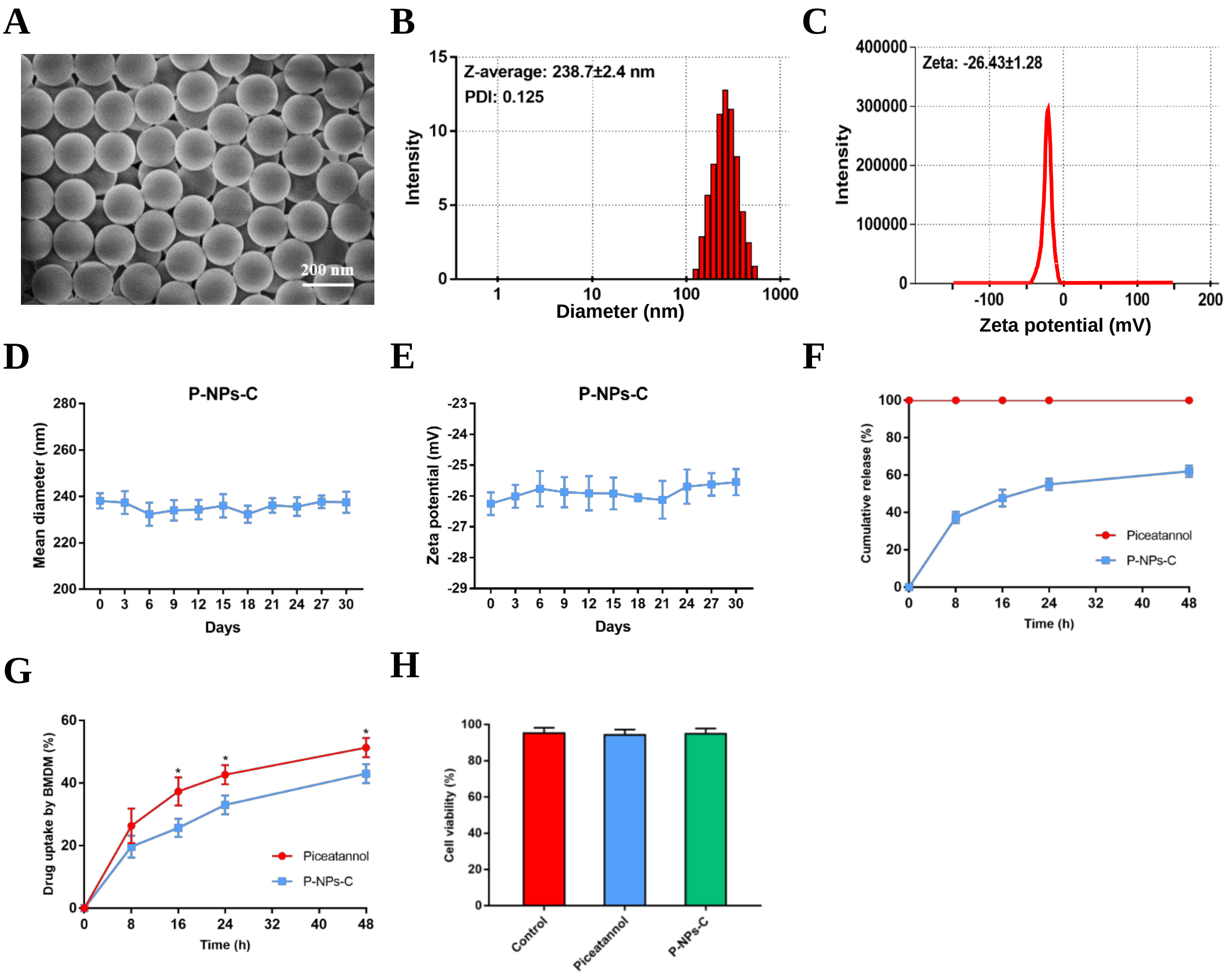
A
200 nm
B
Z-average: 238.7±2.4 nm
PDI: 0.125
Intensity
Diameter (nm)
C
Zeta: -26.43±1.28
Intensity
Zeta potential (mV)
D
P-NPs-C
Mean diameter (nm)
Days
E
P-NPs-C
Zeta potential (mV)
Days
F
Cumulative release (%)
Time (h)
Piceatannol
P-NPs-C
G
Drug uptake by BMDM (%)
Time (h)
Piceatannol
P-NPs-C
H
Cell viability (%)
Control
Piceatannol
P-NPs-C

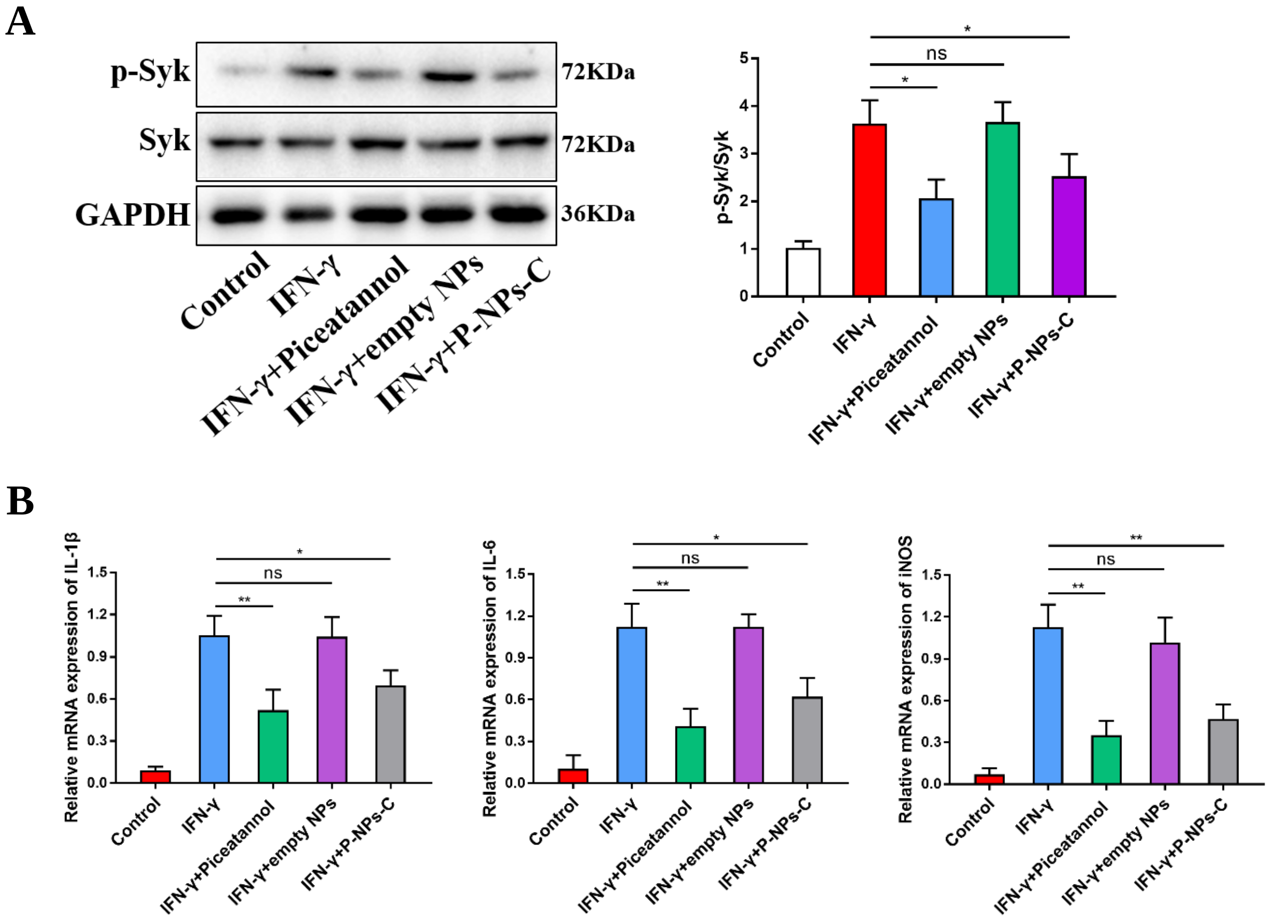
A
p-Syk
Syk
GAPDH
72KDa
72KDa
36KDa
Control
IFN-γ
IFN-γ+Piceatannol
IFN-γ+empty NPs
IFN-γ+P-NPs-C
p-Syk/Syk
0
1
2
3
4
5
*
ns
*
B
Relative mRNA expression of IL-1β
Relative mRNA expression of IL-6
Relative mRNA expression of iNOS
0.0
0.3
0.6
0.9
1.2
1.5
**
ns
*
**
ns
*
**
ns
**

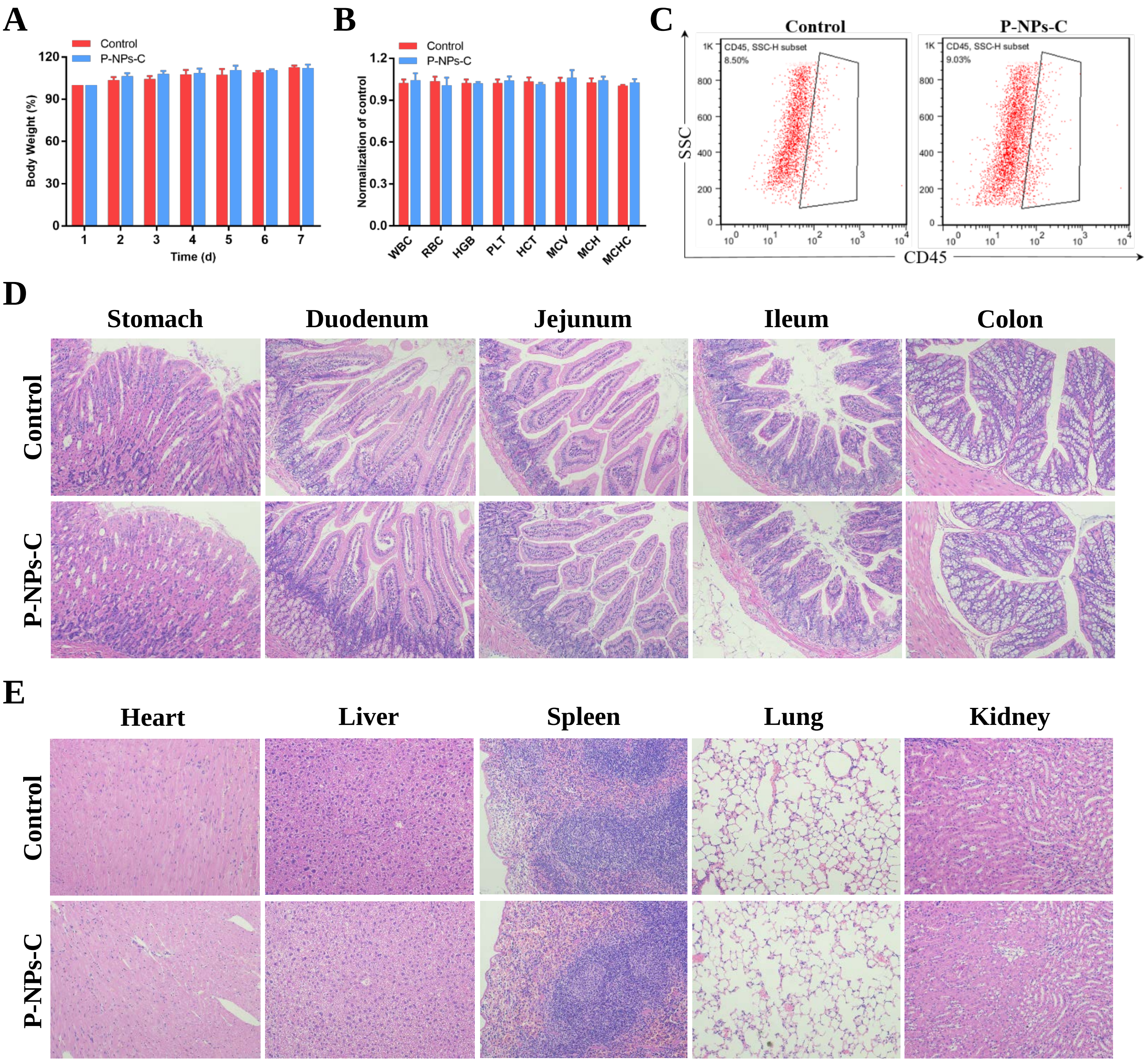

A
Control
P-NPs-C
Body Weight (%)
120
90
60
30
0
1
2
3
4
5
6
7
Time (d)
B
Control
P-NPs-C
Normalization of control
1.2
0.9
0.6
0.3
0.0
WBC
RBC
HGB
PLT
HCT
MCV
MCH
MCHC
C
Control
P-NPs-C
CD45, SSC-H subset
8.50%
9.03%
SSC
CD45
D
Stomach
Duodenum
Jejunum
Ileum
Colon
Control
P-NPs-C
E
Heart
Liver
Spleen
Lung
Kidney
Control
P-NPs-C

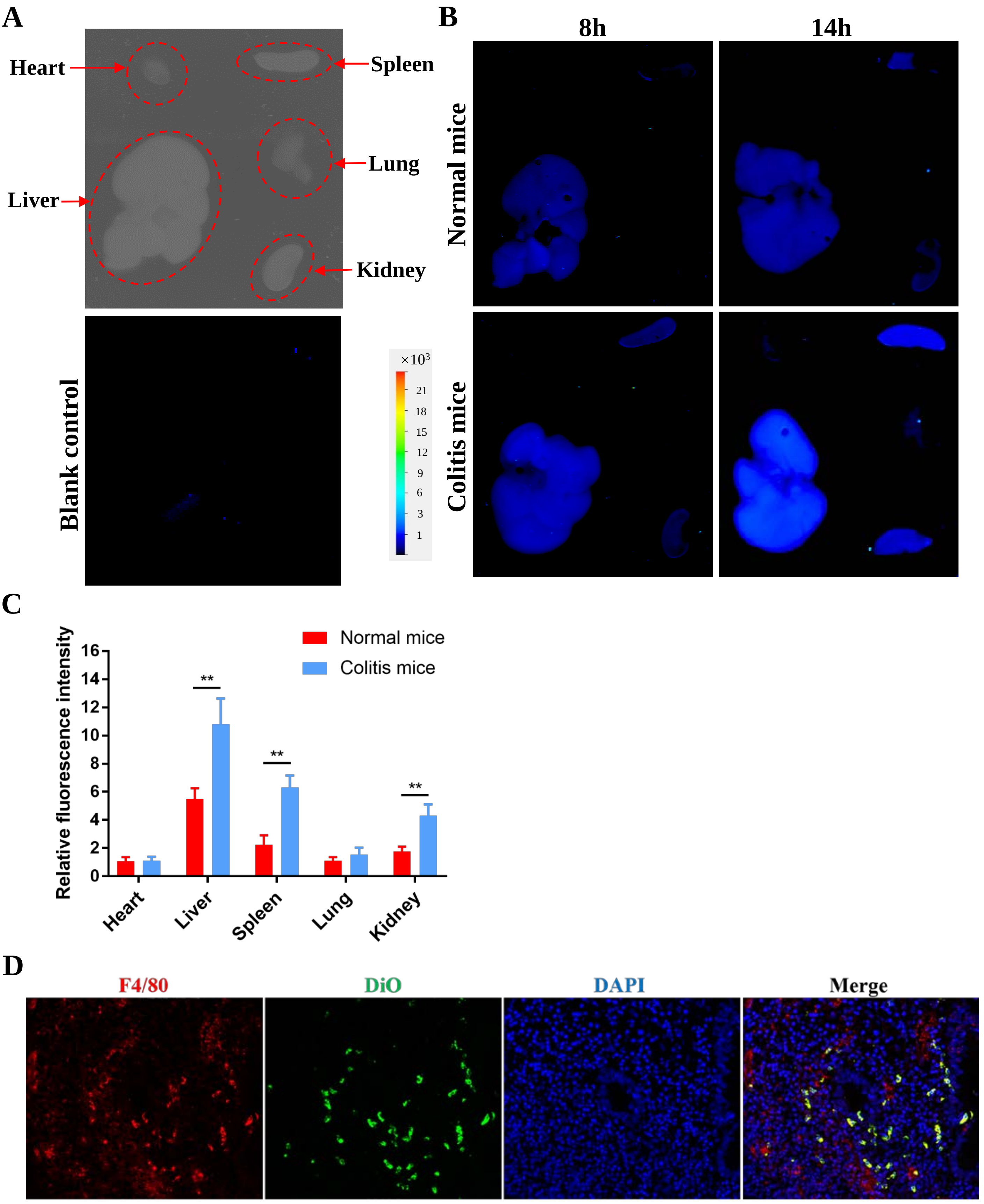
A
Heart
Spleen
Lung
Liver
Kidney
Blank control
×103
21
18
15
12
9
6
3
1
B
8h
14h
Normal mice
Colitis mice
C
Normal mice
Colitis mice
Relative fluorescence intensity
16
14
12
10
8
6
4
2
0
**
**
**
Heart
Liver
Spleen
Lung
Kidney
D
F4/80
DiO
DAPI
Merge

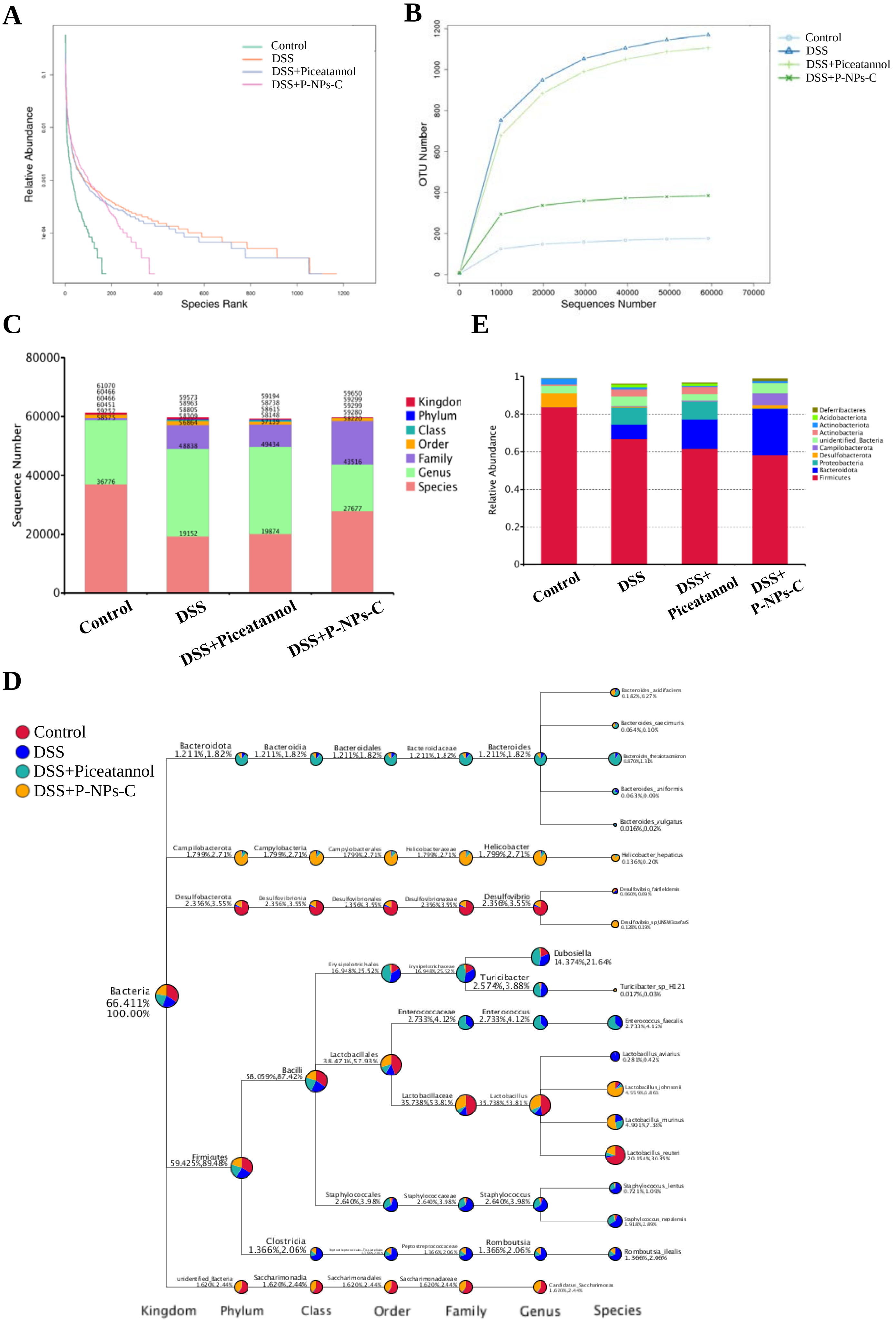
A
Control
DSS
DSS+Piceatannol
DSS+P-NPs-C
Relative Abundance
Species Rank
B
Control
DSS
DSS+Piceatannol
DSS+P-NPs-C
OTU Number
Sequences Number
C
Kingdon
Phylum
Class
Order
Family
Genus
Species
Sequence Number
Control
DSS
DSS+Piceatannol
DSS+P-NPs-C
E
Deferribacteres
Acidobacteriota
Actinobacteriota
Actinobacteria
unidentified_Bacteria
Campilobacterota
Desulfobacterota
Proteobacteria
Bacteroidota
Firmicutes
Relative Abundance
Control
DSS
DSS+ Piceatannol
DSS+ P-NPs-C
D
Control
DSS
DSS+Piceatannol
DSS+P-NPs-C
Bacteria
66.411%
100.00%
Kingdom
Phylum
Class
Order
Family
Genus
Species

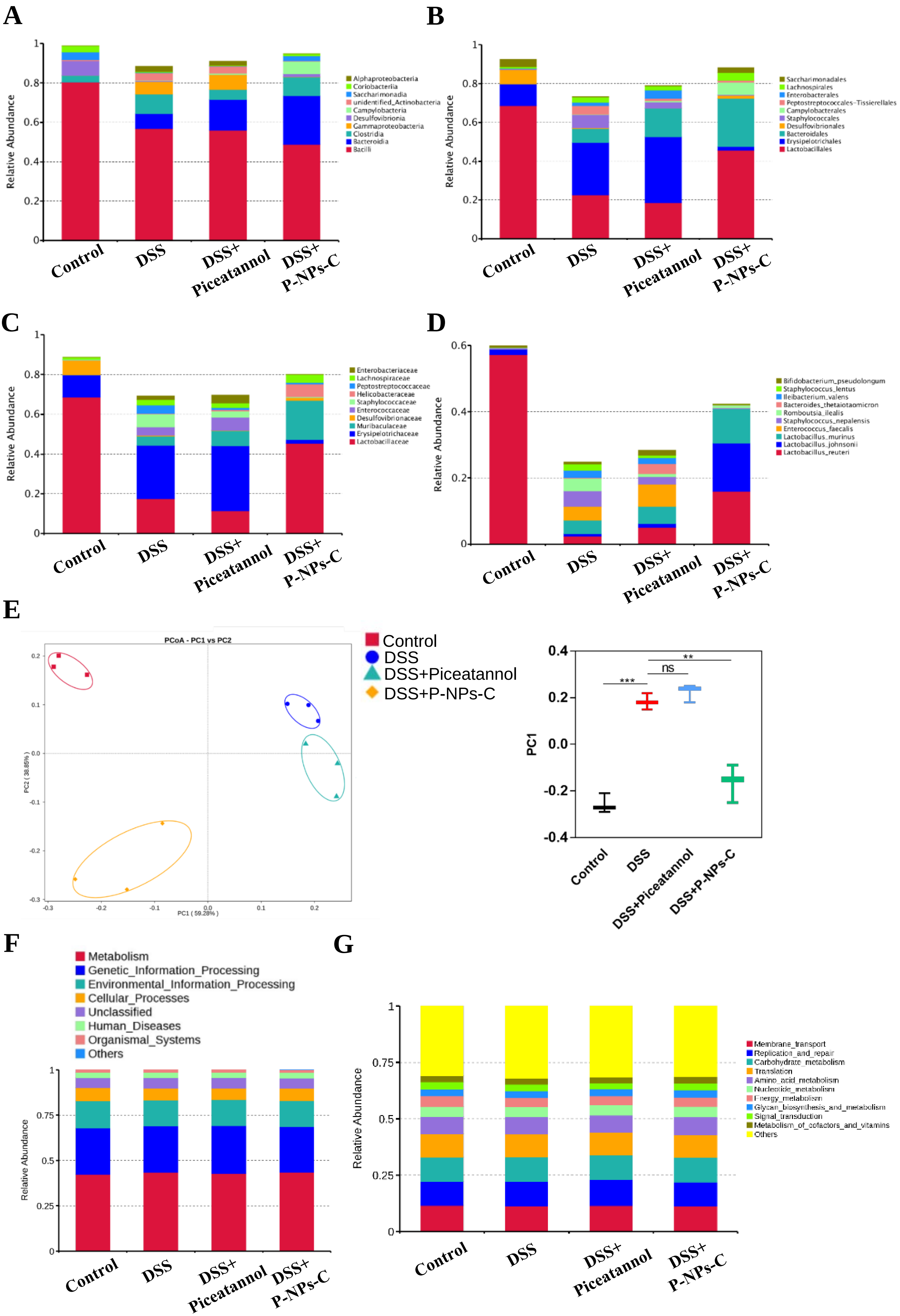
A
Relative Abundance
Alphaproteobacteria
Coriobacteriia
Saccharimonadia
unidentified_Actinobacteria
Campylobacteria
Desulfovibrionia
Gammaproteobacteria
Clostridia
Bacteroidia
Bacilli
Control
DSS
DSS+ Piceatannol
DSS+ P-NPs-C
B
Relative Abundance
Saccharimonadales
Lachnospirales
Enterobacterales
Peptostreptococcales-Tissierellales
Campylobacterales
Staphylococcales
Desulfovibrionales
Bacteroidales
Erysipelotrichales
Lactobacillales
C
Relative Abundance
Enterobacteriaceae
Lachnospiraceae
Peptostreptococcaceae
Helicobacteraceae
Staphylococcaceae
Enterococcaceae
Desulfovibrionaceae
Muribaculaceae
Erysipelotrichaceae
Lactobacillaceae
D
Relative Abundance
Bifidobacterium_pseudolongum
Staphylococcus_lentus
Ileibacterium_valens
Bacteroides_thetaiotaomicron
Romboutsia_ilealis
Staphylococcus_nepalensis
Enterococcus_faecalis
Lactobacillus_murinus
Lactobacillus_johnsonii
Lactobacillus_reuteri
E
PCoA - PC1 vs PC2
PC1 (59.28%)
PC2 (38.85%)
Control
DSS
DSS+Piceatannol
DSS+P-NPs-C
PC1
***
ns
**
F
Metabolism
Genetic_Information_Processing
Environmental_Information_Processing
Cellular_Processes
Unclassified
Human_Diseases
Organismal_Systems
Others
Relative Abundance
G
Relative Abundance
Membrane_transport
Replication_and_repair
Carbohydrate_metabolism
Translation
Amino_acid_metabolism
Nucleotide_metabolism
Energy_metabolism
Glycan_biosynthesis_and_metabolism
Signal_transduction
Metabolism_of_cofactors_and_vitamins
Others

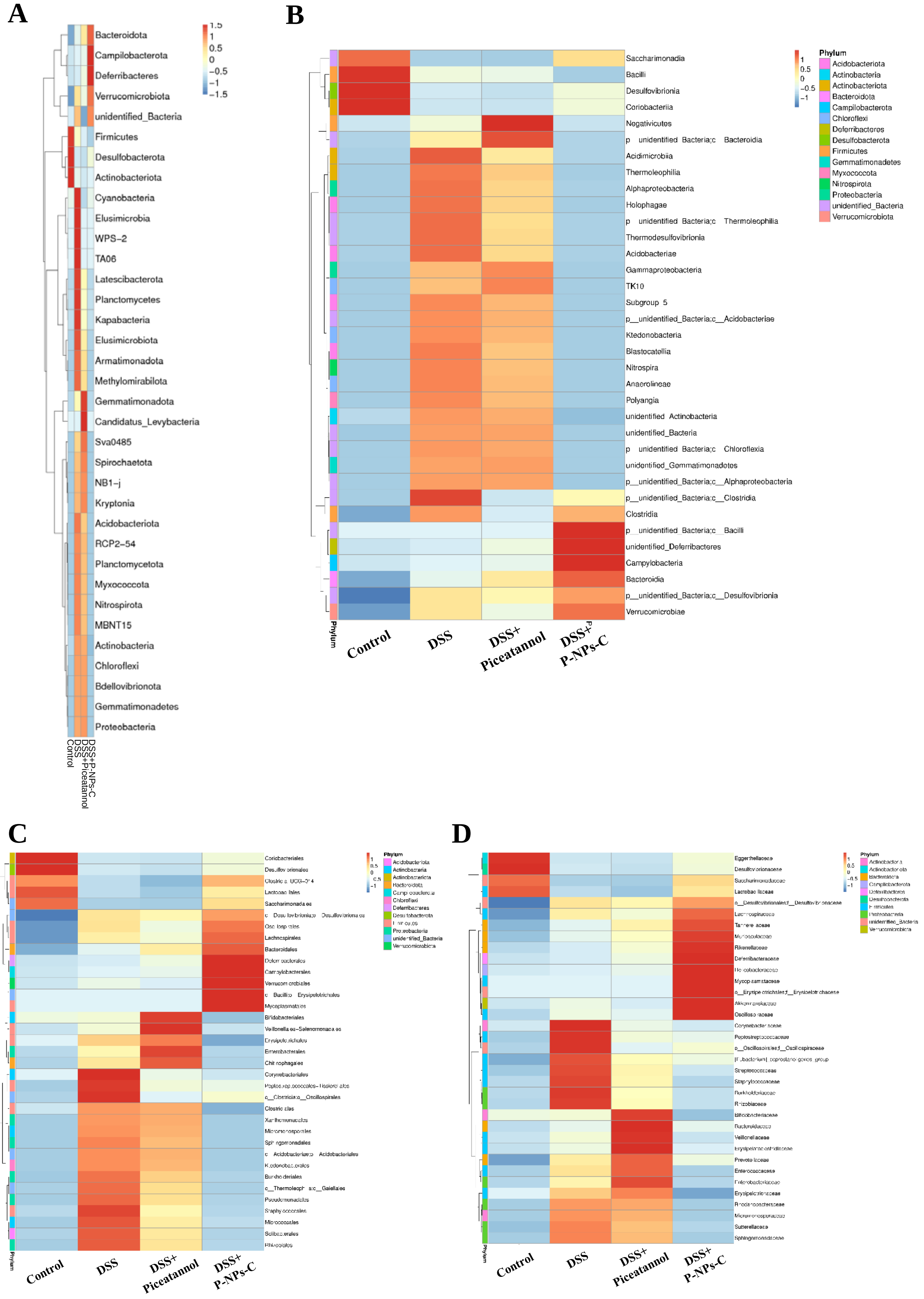
A
Bacteroidota
Campilobacterota
Deferribacteres
Verrucomicrobiota
unidentified_Bacteria
Firmicutes
Desulfobacterota
Actinobacteriota
Cyanobacteria
Elusimicrobia
WPS-2
TA06
Latescibacterota
Planctomycetes
Kapabacteria
Elusimicrobiota
Armatimonadota
Methylomirabilota
Gemmatimonadota
Candidatus_Levybacteria
Sva0485
Spirochaetota
NB1-j
Kryptonia
Acidobacteriota
RCP2-54
Planctomycetota
Myxococcota
Nitrospirota
MBNT15
Actinobacteria
Chloroflexi
Bdellovibrionota
Gemmatimonadetes
Proteobacteria
Control
DSS
DSS+Piceatannol
DSS+P-NPs-C
1.5
1
0.5
0
-0.5
-1
-1.5
B
Saccharimonadia
Bacilli
Desulfovibrionia
Coriobacteriia
Negativicutes
p__unidentified_Bacteria;c__Bacteroidia
Acidimicrobiia
Thermoleophilia
Alphaproteobacteria
Holophagae
p__unidentified_Bacteria;c__Thermoleophilia
Thermodesulfovibrionia
Acidobacteriae
Gammaproteobacteria
TK10
Subgroup_5
p__unidentified_Bacteria;c__Acidobacteriae
Ktedonobacteria
Blastocatellia
Nitrospira
Anaerolineae
Polyangia
unidentified_Actinobacteria
unidentified_Bacteria
p__unidentified_Bacteria;c__Chloroflexia
unidentified_Gemmatimonadetes
p__unidentified_Bacteria;c__Alphaproteobacteria
p__unidentified_Bacteria;c__Clostridia
Clostridia
p__unidentified_Bacteria;c__Bacilli
unidentified_Deferribacteres
Campylobacteria
Bacteroidia
p__unidentified_Bacteria;c__Desulfovibrionia
Verrucomicrobiae
Phylum
Control
DSS
DSS+ Piceatannol
DSS+ P-NPs-C
1
0.5
0
-0.5
-1
Phylum
Acidobacteriota
Actinobacteria
Actinobacteriota
Bacteroidota
Campilobacterota
Chloroflexi
Deferribacteres
Desulfobacterota
Firmicutes
Gemmatimonadetes
Myxococcota
Nitrospirota
Proteobacteria
unidentified_Bacteria
Verrucomicrobiota
C
Coriobacteriales
Lactobacillales
Saccharimonadales
Lachnospirales
Bacteroidales
Campylobacterales
Mycoplasmatales
Bifidobacteriales
Enterobacterales
Corynebacteriales
c__Clostridia;o__Oscillospirales
Xanthomonadales
Sphingomonadales
c__Acidobacteriae;o__Acidobacteriales
Burkholderiales
c__Thermoleophilia;o__Gaiellales
Pseudomonadales
Micrococcales
Solibacterales
Rhizobiales
Phylum
Control
DSS
DSS+ Piceatannol
DSS+ P-NPs-C
Phylum
Acidobacteriota
Actinobacteria
Actinobacteriota
Bacteroidota
Chloroflexi
Deferribacteres
Desulfobacterota
Proteobacteria
unidentified_Bacteria
Verrucomicrobiota
D
Eggerthellaceae
Saccharimonadaceae
Lactobacillaceae
o__Desulfovibrionales;f__Desulfovibrionaceae
Rikenellaceae
Deferribacteraceae
o__Erysipelotrichales;f__Erysipelotrichaceae
Oscillospiraceae
Peptostreptococcaceae
o__Oscillospirales;f__Oscillospiraceae
Streptococcaceae
Staphylococcaceae
Burkholderiaceae
Rhizobiaceae
Bifidobacteriaceae
Veillonellaceae
Prevotellaceae
Enterococcaceae
Erysipelotrichaceae
Sutterellaceae
Sphingomonadaceae
Phylum
Control
DSS
DSS+ Piceatannol
DSS+ P-NPs-C
Phylum
Bacteroidota
Campilobacterota
Deferribacteres
Desulfobacterota
Firmicutes
Proteobacteria
unidentified_Bacteria
Verrucomicrobiota

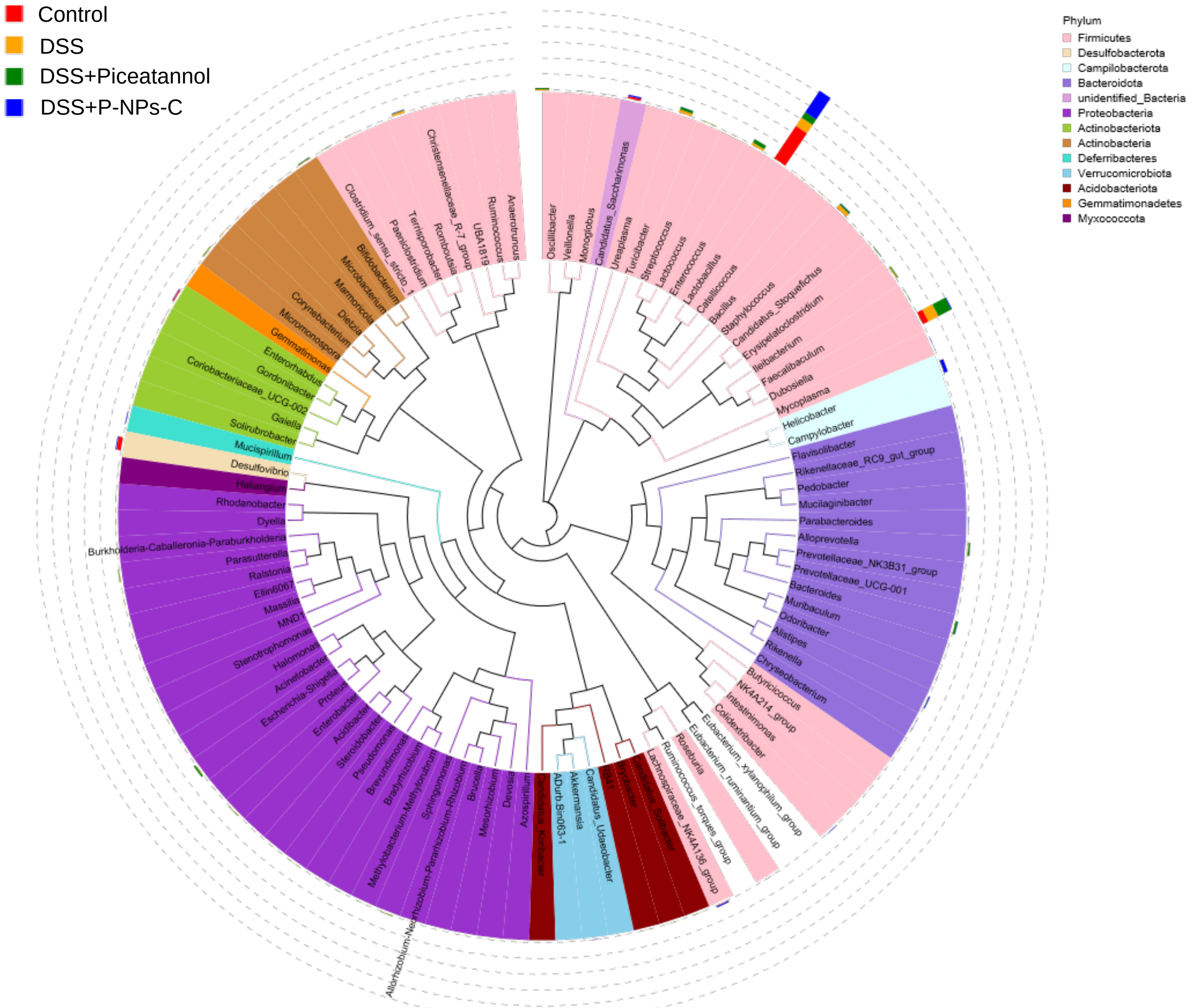
Control
DSS
DSS+Piceatannol
DSS+P-NPs-C
Phylum
Firmicutes
Desulfobacterota
Campilobacterota
Bacteroidota
unidentified_Bacteria
Proteobacteria
Actinobacteriota
Actinobacteria
Deferribacteres
Verrucomicrobiota
Acidobacteriota
Gemmatimonadetes
Myxococcota
Oscillibacter
Veillonella
Monoglobus
Candidatus_Saccharimonas
Ureaplasma
Turicibacter
Streptococcus
Lactococcus
Enterococcus
Lactobacillus
Catellicoccus
Bacillus
Staphylococcus
Candidatus_Stoquefichus
Erysipelatoclostridium
Ileibacterium
Faecalibaculum
Dubosiella
Mycoplasma
Helicobacter
Campylobacter
Flavisolibacter
Rikenellaceae_RC9_gut_group
Pedobacter
Mucilaginibacter
Parabacteroides
Alloprevotella
Prevotellaceae_NK3B31_group
Prevotellaceae_UCG-001
Bacteroides
Muribaculum
Odoribacter
Alistipes
Rikenella
Chryseobacterium
Butyricicoccus
NK4A214_group
Intestinimonas
Colidextribacter
Eubacterium_xylanophilum_group
Eubacterium_ruminantium_group
Roseburia
Ruminococcus_torques_group
Lachnospiraceae_NK4A136_group
Candidatus_Solibacter
Candidatus_Udaeobacter
Akkermansia
ADurb.Bin063-1
Candidatus_Koribacter
Azospirillum
Devosia
Mesorhizobium
Brucella
Allorhizobium-Neorhizobium-Pararhizobium-Rhizobium
Sphingomonas
Methylobacterium-Methylorubrum
Bradyrhizobium
Brevundimonas
Pseudomonas
Steroidobacter
Acidibacter
Enterobacter
Proteus
Escherichia-Shigella
Acinetobacter
Halomonas
Stenotrophomonas
MND1
Massilia
Ellin6067
Ralstonia
Parasutterella
Burkholderia-Caballeronia-Paraburkholderia
Dyella
Rhodanobacter
Haliangium
Desulfovibrio
Mucispirillum
Solirubrobacter
Gaiella
Coriobacteriaceae_UCG-002
Gordonibacter
Enterorhabdus
Gemmatimonas
Micromonospora
Corynebacterium
Dietzia
Marmoricola
Microbacterium
Bifidobacterium
Clostridium_sensu_stricto_1
Paeniclostridium
Terrisporobacter
Romboutsia
Christensenellaceae_R-7_group
UBA1819
Ruminococcus
Anaerotruncus

Supplementary table 1. Demographic and main characteristics of the study population

	CD	UC	Controls
Number, n	17	13	10
Median age, yr, (IQR)	32.7 (20.8-52.4)	41.0 (26.3-49.1)	34.8 (24.1-57.3)
Men, n (%)	10 (58.8)	6 (46.2)	6 (60.0)
Origin of samples, n (%)			
Ileum	15 (29.4)	0	5 (21.7)
Colon	36 (70.6)	29 (100.0)	18 (78.3)
Simple CDAI score (Mean ±SEM)	12.3±1.2	-	-
Modified Mayo score (Mean ±SEM)	-	6.4±0.7	-
Disease severity, n (%)			
No	-	-	10 (100.0)
Mild	4 (23.5)	5 (38.5)	-
Moderate	9 (53.0)	7 (53.8)	-
Severe	4 (23.5)	1 (7.7)	-
Median disease duration, mon, (IQR)	15.6 (3.7-39.5)	13.3 (4.6-22.8)	-
Disease location, n (%)			
L1 or E1	5 (29.4)	2 (15.4)	-
L2 or E2	8 (47.1)	7 (53.8)	-
L3 or E3	4 (23.5)	4 (30.8)	-
Disease behavior, n (%)			
B1	12 (70.6)	-	-
B2	4 (23.5)	-	-
B3	1 (5.9)	-	-
Perianal lesions, n (%)	3 (17.6)	-	-

Supplementary table 2. Primer sequences.

Gene Name	Species	Sequence
Syk	human	5'-CATGGAAAAATCTCTCGGGAAGA-3'<br>5'-GTCGATGCGATAGTGCAGCA-3'
IL-1β	mouse	5'-ATGATGGCTTATTACAGTGGCAA-3'<br>5'-GTCGGAGATTCGTAGCTGGA-3'
IL-6	mouse	5'-TAGTCCTTCCTACCCCAATTTCC-3'<br>5'-TTGGTCCTTAGCCACTCCTTC-3'
iNOS	mouse	5'-GGTGAAGGGACTGAGCTGTT-3'<br>5'-GCTACTCCGTGGAGTGAACAA-3'
CCR5	mouse	5'-TTTTCAAGGGTCAGTTCCGAC-3'<br>5'-GGAAGACCATCATGTTACCCAC-3'
CARD9	mouse	5'-GGCTCTGAACAAGGAGCATCT-3'<br>5'-TCCTGCGGTAGTTCTCGAAG-3'
TNF-α	mouse	5'-AGGCTGCCCCGACTACGT-3'<br>5'-GACTTTCTCCTGGTATGAGATAGCAAA-3'
